# Novel stochastic framework for automatic segmentation of human thigh MRI volumes and its applications in spinal cord injured individuals

**DOI:** 10.1371/journal.pone.0216487

**Published:** 2019-05-09

**Authors:** Samineh Mesbah, Ahmed M. Shalaby, Sean Stills, Ahmed M. Soliman, Andrea Willhite, Susan J. Harkema, Enrico Rejc, Ayman S. El-Baz

**Affiliations:** 1 Department of Electrical and Computer Engineering, University of Louisville, Louisville, KY, United States of America; 2 Department of Bioengineering, University of Louisville, Louisville, KY, United States of America; 3 Kentucky Spinal Cord Injury Research Center, University of Louisville, Louisville, KY, United States of America; 4 Department of Neurological Surgery, University of Louisville, Louisville, KY, United States of America; 5 Frazier Rehab Institute, Kentucky One Health, Louisville, KY, United States of America; Center for Neuroscience and Regenerative Medicine, UNITED STATES

## Abstract

Severe spinal cord injury (SCI) leads to skeletal muscle atrophy and adipose tissue infiltration in the skeletal muscle, which can result in compromised muscle mechanical output and lead to health-related complications. In this study, we developed a novel automatic 3-D approach for volumetric segmentation and quantitative assessment of thigh Magnetic Resonance Imaging (MRI) volumes in individuals with chronic SCI as well as non-disabled individuals. In this framework, subcutaneous adipose tissue, inter-muscular adipose tissue and total muscle tissue are segmented using linear combination of discrete Gaussians algorithm. Also, three thigh muscle groups were segmented utilizing the proposed 3-D Joint Markov Gibbs Random Field model that integrates first order appearance model, spatial information, and shape model to localize the muscle groups. The accuracy of the automatic segmentation method was tested both on SCI (N = 16) and on non-disabled (N = 14) individuals, showing an overall 0.93±0.06 accuracy for adipose tissue and muscle compartments segmentation based on Dice Similarity Coefficient. The proposed framework for muscle compartment segmentation showed an overall higher accuracy compared to ANTs and STAPLE, two previously validated atlas-based segmentation methods. Also, the framework proposed in this study showed similar Dice accuracy and better Hausdorff distance measure to that obtained using DeepMedic Convolutional Neural Network structure, a well-known deep learning network for 3-D medical image segmentation. The automatic segmentation method proposed in this study can provide fast and accurate quantification of adipose and muscle tissues, which have important health and functional implications in the SCI population.

## Introduction

Spinal cord injury (SCI) is one of the primary causes of motor disabilities in humans, with an annual incidence of approximately 17700 new cases only in the United States [1]. Skeletal muscles experience deleterious physiological changes after SCI. Animal studies showed that spinal cord transection reduced muscle mass of hind-limb extensors between 20% and 40% in one month [2–4]. Individuals with chronic SCI also showed cross-sectional area of the whole thigh, knee extensors and plantar flexors that were about 30% smaller compared to non-disabled individuals [5, 6]. In addition, SCI leads to fat infiltration within the muscle (intramuscular adipose tissue) and between muscles (intermuscular adipose tissue, IMAT) [6, 7]. The consequences of severe SCI-induced adaptations on skeletal muscle are two-fold: on one side, the muscle mechanical output is compromised, both in terms of force exertion and fatigue resistance [8, 9], which could limit motor function even if the recovery of neural control was sufficient. Additionally, the concurrent loss of muscle tissue and gain of ectopic (i.e. non-subcutaneous) adipose tissue can favor health-related complications such as pressure ulcer, glucose intolerance, insulin resistance and therefore type II diabetes, metabolic syndrome and cardiovascular disease [6, 7]. However, these negative muscle adaptations can be mitigated by proper interventions that include neuromuscular electrical stimulation, dietary programs and assisted movement trainings [10, 11].

Magnetic resonance imaging (MRI) is a suitable method for examining the effects of SCI and determining the effectiveness of subsequent rehabilitative interventions on skeletal muscle and adipose tissue distribution, because of its multiple forms of contrast weighting and its sensitivity to diffusion, perfusion, and chemical composition of tissues [12]. In particular, determining the SCI- and intervention-induced adaptations in functional key muscle groups, such as knee extensors and flexors, is important for understanding if appropriate muscle volume and ratio between key muscle groups are present at different stages post SCI, as this can affect motor function. Also, MRI can provide useful information for understanding ectopic adipose tissue-related adaptations after SCI and different interventions in order to optimize prevention of SCI-induced health complications. For example, individual characteristics of SCI such as the level of spasticity may influence ectopic adipose tissue distribution as well as skeletal muscle size [13]. Similarly, it is important to evaluate the effects of different interventions (i.e. dietary planning; activity-based training) in order to understand their efficacy and select the most appropriate ones for reducing ectopic adipose tissue and thus contributing to prevent SCI-induced health complications.

It is important to recognize that manually assessing these parameters from MR images presents some relevant limitations, as manual segmentation is laborious, time-consuming, impractical for large studies, and can make the estimated indices subjective to the inter-rater variability to some extent. To overcome these issues, different automatic segmentation methods of MRI images were proposed in the literature. Initial attempts for automatic segmentation of the MR thigh images started with the work of Barra et al. [14, 15] where they used intensity differences between tissues to segment the total muscle and fat areas in elderly population and it was further advanced by others to segment subcutaneous adipose tissue (SAT), IMAT and bone areas in obese or elderly populations [16–18]. More recently, various algorithms were proposed to use prior shape information to segment individual thigh muscles in healthy and elderly populations and individuals with chronic diseases [19, 20]. Automatic and semi-automatic atlas-based methods using image registration algorithms for muscle segmentation have gained more attention recently to segment quadriceps individual muscles and muscle group in healthy individuals [21, 22]. There have also been longitudinal studies on the human thigh muscles using the semi-automatic segmentation methods in individuals with osteoarthritis and those with muscular dystrophy [23, 24].

The field of segmentation and quantitative assessments of medical images has been considerably impacted in recent years by the emergence of deep learning neural networks. In particular, two and three-dimensional Convolutional Neural Networks (CNNs) have shown promising results in various medical imaging fields including several recent studies on segmentation of bone and cartilage in MRI thigh and knee scans [25–29]. However, there are several limitations and additional requirements needed for the application of CNNs on 3-D medical images segmentation, such as an increased number of adjustable network parameters, substantial memory and computational costs, and the need of high-level engineering expertise to properly perform training and testing steps for pre-designed networks [26, 30–32].

Despite the relevant developments regarding the automatic segmentation and quantitative assessments of medical images that have been reported in the literature, there has been no explicit automatic segmentation framework proposed for the SCI population that can accurately and efficiently segment muscle groups like knee extensors and flexors, which play a key functional role, and quantify different types of adipose tissue such as SAT and IMAT. In the present study, we propose a novel stochastic method that integrates intensity, spatial information and shape model to separate fat volumes from the muscle tissue and segment the muscle tissue into three compartments (knee extensors, knee flexors and a medial compartment including the adductor muscles), utilizing the Joint Markov Gibbs Random Field (MGRF) model. In order to test the accuracy of this method, we have applied the proposed framework on both a group of individuals with chronic SCI and a group of non-disabled (ND) individuals, comparing the automatic segmentation outcomes to those obtained from manual segmentation. Moreover, we have also compared the outcomes generated from our novel three-fold stochastic method with those obtained from two other well-known atlas-based techniques in order to highlight the potential improvements brought about by our approach. Finally, the thigh muscle and fat segmentation task was also performed using a well-known CNN architecture to evaluate the advantages and limitations of deep learning approach for this application.

## Materials and methods

### MRI scan specifications and characteristics of the research participants

In this work, the 3-D MRI scans were acquired using Siemens 3T Magnetom Skyra with pulse sequence–t1 vibe (for 3-D VIBE images) for in phase, opposite phase, water, and fat imaging. The volume dimensions (X, Y, Z) are 320 by 208 by 320 and the series length is 1. Voxel dimensions (X, Y, Z) are 1.5 x 1.5 x 1.5 mm, size of series point is 0.006 seconds and the slice gap is equal to zero. The thigh MRI scans analyzed in this study were collected from a total of 30 participants including 16 individuals with chronic SCI and 14 ND subjects. The characteristics of the 16 individuals with severe chronic SCI were the following: age (year): 32.4 ± 9.1; time since injury (year): 6.7 ± 7.7; 13 males and 3 females; 10 individuals classified as American Spinal Injury Association (ASIA) impairment scale (AIS) A, 5 individuals as AIS B, and 1 individual as C as for the International Standards for Neurological Classification of Spinal Cord Injury [33]; height (m): 1.78 ± 0.09; weight (kg): 77.19 ± 12.48; body mass index (BMI) (kg/m^2^): 24.36 ± 3.99. The 14 ND subjects included in this study presented were 11 males and 3 females with age (year): 28.47 ± 3.8; height (m): 1.80 ± 0.10; weight (kg): 92.56 ± 15.30; BMI (kg/m^2^): 28.54 ± 4.27 (S1 Table). All participants were fully informed about the aims of the study and written consent was provided from all individuals, which was approved by the University of Louisville Institutional Review Board. All research activities were performed in accordance with the guidelines and regulations of this Institutional Review Board.

### Automatic segmentation framework

**Fig 1:**
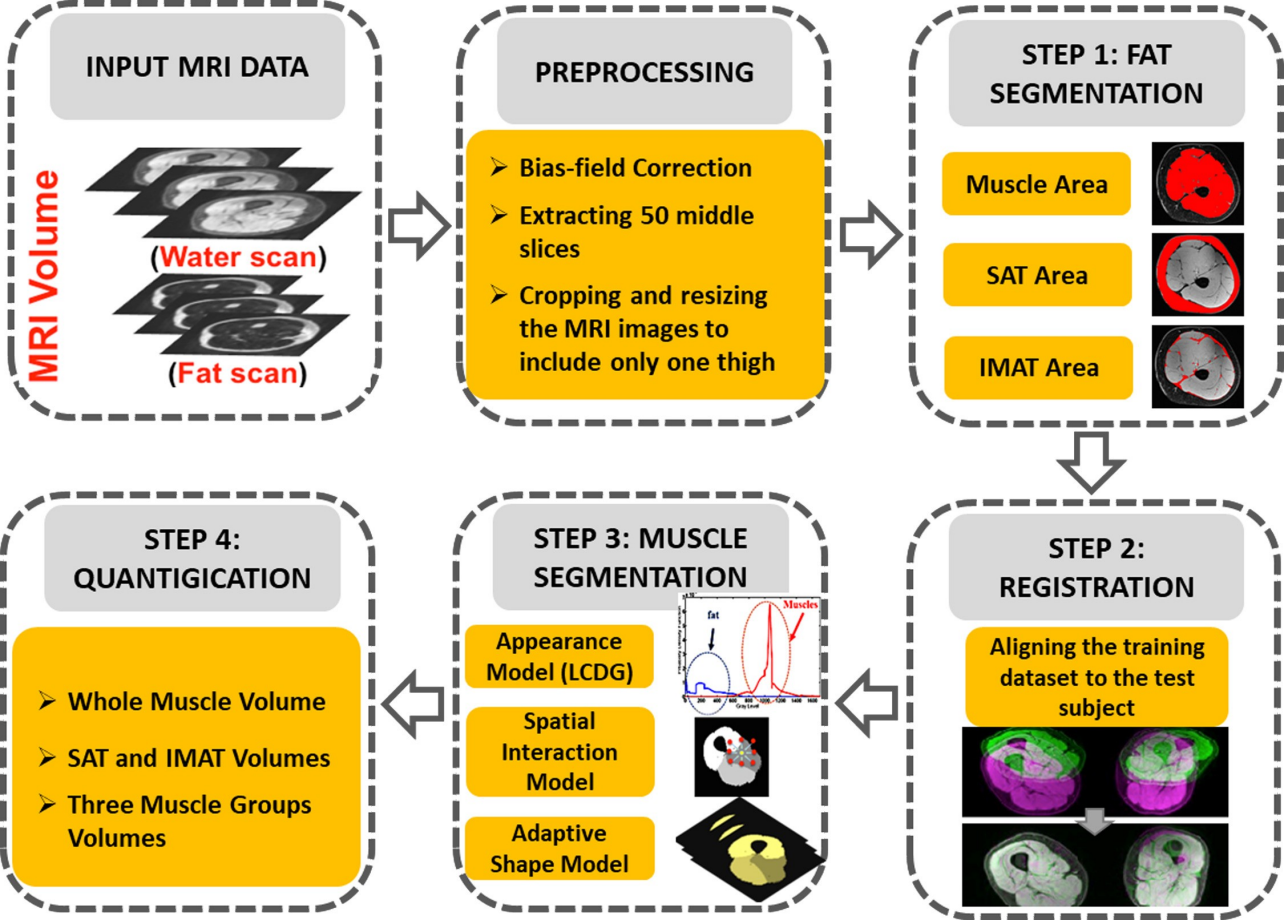
The proposed framework. The Block diagram of the proposed framework for muscles/fat segmentation and quantification based on MRI 3-D volumes.

A 3-D stochastic framework for fat suppressed (FS) and water suppressed (WS) MRI muscles and fat segmentation is proposed in Fig 1. The proposed system consists of a preprocessing step to prepare the data for automatic segmentation, which includes bias-field correction, extraction of the central 50 slices between greater trochanter and lateral epicondyle of the femur, and cropping and resizing the MRI images to include only one thigh for further processing steps. The automatic segmentation part is divided to 4 steps. In the first step, which is devoted to fat and muscle area segmentation, the sum of WS and FS volumetric MRI is utilized to get the mask of the whole thigh volume and the bone marrow area utilizing Linear Combination of Discrete Gaussians (LCDG) algorithm [34]. The same method was used on each FS-MRI volume to initially extract muscle volume and WS-MRI volume to segment the total adipose tissue. Moreover, SAT was separated from IMAT by overlaying the muscle tissue mask, obtained from the FS volume, on the total fat segments from the WS volume. In the second step, each greyscale muscle volume and its manually segmented muscle groups (training dataset) are co-aligned to a reference dataset using a 3-D cubic B-splines-based approach (described in [35]) to account for the anatomical differences of each patient’s extracted muscle volumes. The third step consists of joint Markov model that simultaneously maximizes the likelihood estimation of three components: Appearance-based shape (muscles anatomy), spatial (second order appearance) and intensity (first order appearance) models by using iterated conditional modes to localize and segment three muscle groups (knee extensors, knee flexors and the medial compartment, which includes Sartorius, adductor longus, gracilis, adductor brevus, and adductor magnus muscles) for the test subjects. The fourth and last step consisted in quantifying the effects of SCI on human thigh muscles by calculating the volume of the segmented tissues. More details about the joint Markov model will be discussed in the following sections.

#### Linear Combination of Discrete Gaussians (LCDG)

**Fig 2:**
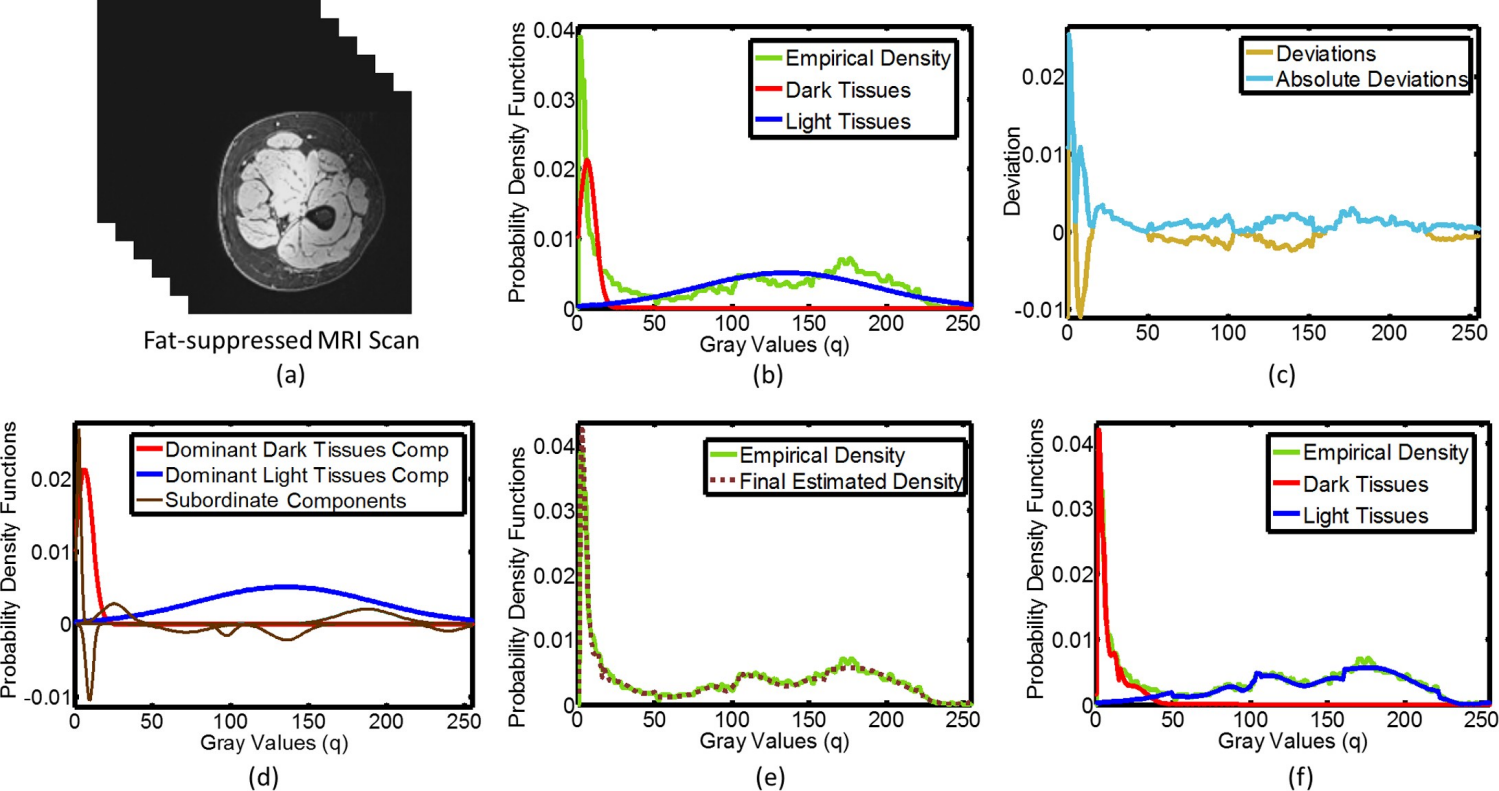
An Example for applying LCDG algorithm MRI 3-D volumes. LCDG algorithm output on (a) exemplary 3D FS-MRI image data; (b) probability density functions of the image voxels in Fig 2A, as determined empirically, and as approximated via LCDG using two dominant DGs; (c) the deviations (standard and absolute) between the empirical and estimated marginal probability density functions in Fig 2B; (d) LCDG algorithm output on the dominant and subordinate DGs in the image data in Fig 2A; (e) the final estimated LCDG model of the empirical density function; and (f) the final LCDG output of the conditional probability density functions of light tissue (muscle) and dark tissue (fat) intensities and the empirical density function.

The main objective of LCDG is to find the threshold for each gray volume that extracts the 2 classes (corresponding to the dark tissues, and light tissues) from their background. In case of FS-MRI scans (Fig 2A), the dark tissues represent the fat and the light tissues represent the muscles area (vice versa for WS-MRI scans). At the end of the LCDG step, we get two probabilities for each voxel of the input volumes: P1 which is the probability of belonging to class 1 (dark tissue) and P2 which is the probability of belonging to class 2 (light tissue). The voxel-wise LCDG probability, will be combined to obtain the muscle, SAT and IMAT areas from FS, WS and FS+WS MRI scans. Fig 2 illustrates the steps of the muscle area segmentation using LCDG algorithm for FS-MRI volumes. Fig 2B shows the initial approximation of the bi-modal empirical distribution of Q = 256 grey levels over a typical FS-MRI volume of human thigh. The dominant modes represent the brighter muscles area and its darker background (fat area). After calculating the deviations between the dominant modes and the empirical distribution, the additive and subtractive parts of the deviations (standard and absolute) are approximated with DG mixtures. The initial mixed LCDG-model consists of the 2 dominant, 4 additive and 4 subtractive DGs (brown curves), as shown in Fig 2C and 2D). Finally, the estimated LCDG as well as conditional LCDG models of the two classes (i.e. muscles and fat tissues) are illustrated in Fig 2E and 2F. This algorithm is also used for WS-MRI scans to segment the fat tissue and FS+WS to segment the whole thigh mask and the bone.

#### Joint Markov Gibbs Random Field Model

In order to divide the extracted muscles area into various groups, a registered-to-reference database of grayscale volume, **g**, of the muscle groups area and its map, **m**, are described with a joint probability model: *P*(**g**,**m**) = *P*(**g**|**m**)*P*(**m**), which combines a conditional probability distribution of the input volume given the map *P*(**g**|**m**), and an unconditional probability distribution of maps P(**m**) = *P*_*sp*_(**m**)*P*_***V***_(**m**), where, *P*_*sp*_(**m**) represents an adaptive shape prior and *P*_***V***_(**m**) is a Gibbs probability distribution with potentials **V**, which denotes a sample of a 3D MGRF model of **m** [36].

**Appearance-based shape model.** In order to reduce the variability across subjects and enhance the segmentation accuracy, an adaptive shape model of each muscle group is employed. To create the shape database, a selected training set of volumes, collected from manually segmented subjects, are registered to a reference dataset using a 3-D B-splines-based transformation that is previously developed [35]. The selection for training dataset has been done based on the 2-D correlation coefficient (number between 0 and 1) between the grayscale images of the manually segmented volumes and the test volume. If the average correlation coefficient for the whole volume is more than or equal to 0.5, that dataset will be selected for training otherwise it will be rejected. After selection, the training volumes are registered to the reference volume. Therefore, for each new test subject, an individual training set is built to help with variability reduction for muscle group segmentation.

In summary, each source volume ***f*** (i.e., each of the training subjects) is aligned to the reference template ***g*** on a domain Ω ⊂ *R*^*3*^ by using a non-rigid registration. Given a certain source ***f***, the registration estimates the deformation field ***T*** for all *x* ∈ Ω, by displacing a sparse grid, Ω’ ⊂ Ω of control points, ζ:

$$T\left(x\right)=x+\underset{\zeta \epsilon \Omega^{,}}{\sum }\zeta \left(\left\|x-\zeta \right\|\right)\Delta \zeta$$ (1)

where Δζ is the displacement vector of the control point ζ and the weighting function ζ (.) measures the contribution of any control point in Ω’ to the displacement of a point in Ω. The goal is that the deformation field minimizes the point-wise dissimilarity between the target ***g*** and the deformed source ***f***:

$$E\left(T\right)=\frac{1}{\left|\mathrm{\Omega '}\right|}+\underset{\zeta \epsilon \Omega^{,}}{\sum }\frac{\phi (g\left(x\right),f\left(T\left(x\right)\right)}{\zeta \left(\left\|x-\zeta \right\|\right)}dx$$ (2)

where ϕ is the dissimilarity function (we used the sum of absolute differences). The objective function in Eq 2 is minimized using a Markov random field model of displacements of the control points ζ [36]. The dense displacement field is then determined from the control point displacements through representing free form deformations via cubic B-splines. We have selected this method because it is fully automated (no manual initialization or hand-picked landmarks) and has low computational time. More details can be found in [35], [36].

The probabilistic shape priors are spatially variant independent random fields of region labels, as following:

$$P_{sp}\left(m\right)\;=\;\prod \;p_{sp:x,y,z}\left(m_{x,y,z}\right)$$ (3)

where *p*_sp:*x*,*y*,*z*_(*l*) is the voxel-wise empirical probabilities for each label *l* ∈ **L**. To segment each input MRI data, an adaptive process guided by the visual appearance features of the input MRI data is used to construct the shape prior. This shape prior consists of four labels: the 3 muscle groups and the background. In the training phase, we use N-1 (N number of subjects) manually segmented data sets by an MRI expert to create the probabilistic maps for the four labels. For the testing phase, each training set is registered using the same approach in [35], to the test MRI volume used to create the discussed shape prior.

**Spatial interaction (second-order appearance model).** In order to overcome noise effects and to ensure segmentation homogeneity, spatially homogeneous 3D pair-wise interactions between the region labels are additionally incorporated in the proposed segmentation model. These interactions are estimated using the Potts model, i.e., an MGRF with the nearest 26-neighbors of the voxels (also known as cliques), and analytic bi-valued Gibbs potentials, that depend only on whether the nearest pairs of labels are equal or not. The utilized second-order 3D MGRF model of the region map **m** is defined as:

$$P_{V}\left(m\right)=\frac{1}{Z_{v_{s}}}\exp\underset{\left(x,y,z\right)\in R}{\sum }\underset{\left(x^{\prime },y^{\prime },z^{\prime }\right)\in v_{s}}{\sum }\text{V}\left(m_{x,y,z},m_{x+x^{\prime },y+y^{\prime },z+z^{\prime }}\right)$$ (4)

where $Z_{v_{s}}$ is the normalization factor. Let *f*_eq_(**m**) denote the relative frequency of equal labels in the neighboring voxel pairs. The initial region map results in an approximation with the following analytical maximum likelihood estimates of the potentials [37]:

$$v_{eq}=\;-v_{ne}\approx \;2f_{eq}\left(m\right)\;–\;1$$ (5)

which allows for computing the voxel-wise probabilities $\;p_{V:x,y,z}(l)$ of each label; *l* ∈ **L**. More details are in [34].

**Intensity (first-order appearance) model.** Our approach also accounts for the visual appearance of the muscles besides the learned shape model and the spatial interactions. Therefore, an intensity-based model using LCDG with positive and negative DG sub-components is applied to improve the initially obtained segmentation accuracy. The role of LCDG is to accurately approximate the empirical gray level distribution of FS-MRI voxel intensities with combination of dominant and subordinate DGs for each label (muscle group). This approximation adapts the segmentation to the changes in appearance, such as non-linear intensity variations caused by different IMAT distributions between muscle groups. At the end of this stage, each grayscale voxel existing in the target volume was mapped to a class with the highest occurrence probability.

**Algorithm I: The proposed muscles group segmentation approach**

For each input FS and WS MRI volumes with grayscale volume g:

             a) Use LCDG to initially extract muscle volume from adipose tissue and bone.
             b) Select atlas volumes using 2-D correlation coefficient measure between the training database and the target volume.
             c) Use non-linear registration to transpose selected atlas volumes' voxels to the reference volume space.
             d) Form an initial region map m using the marginal estimated density and prior shape of each muscle group label.
             e) Find the Gibbs potentials for the MGRF model from the initial map.
             f) Approximate the marginal intensity distribution P(g|m) of each muscle group using LCDG.
             g) Improve the region map m by assigning each voxels to a class with the highest probability density based on its gray value.

### Segmentation accuracy metrics

**Fig 3:**
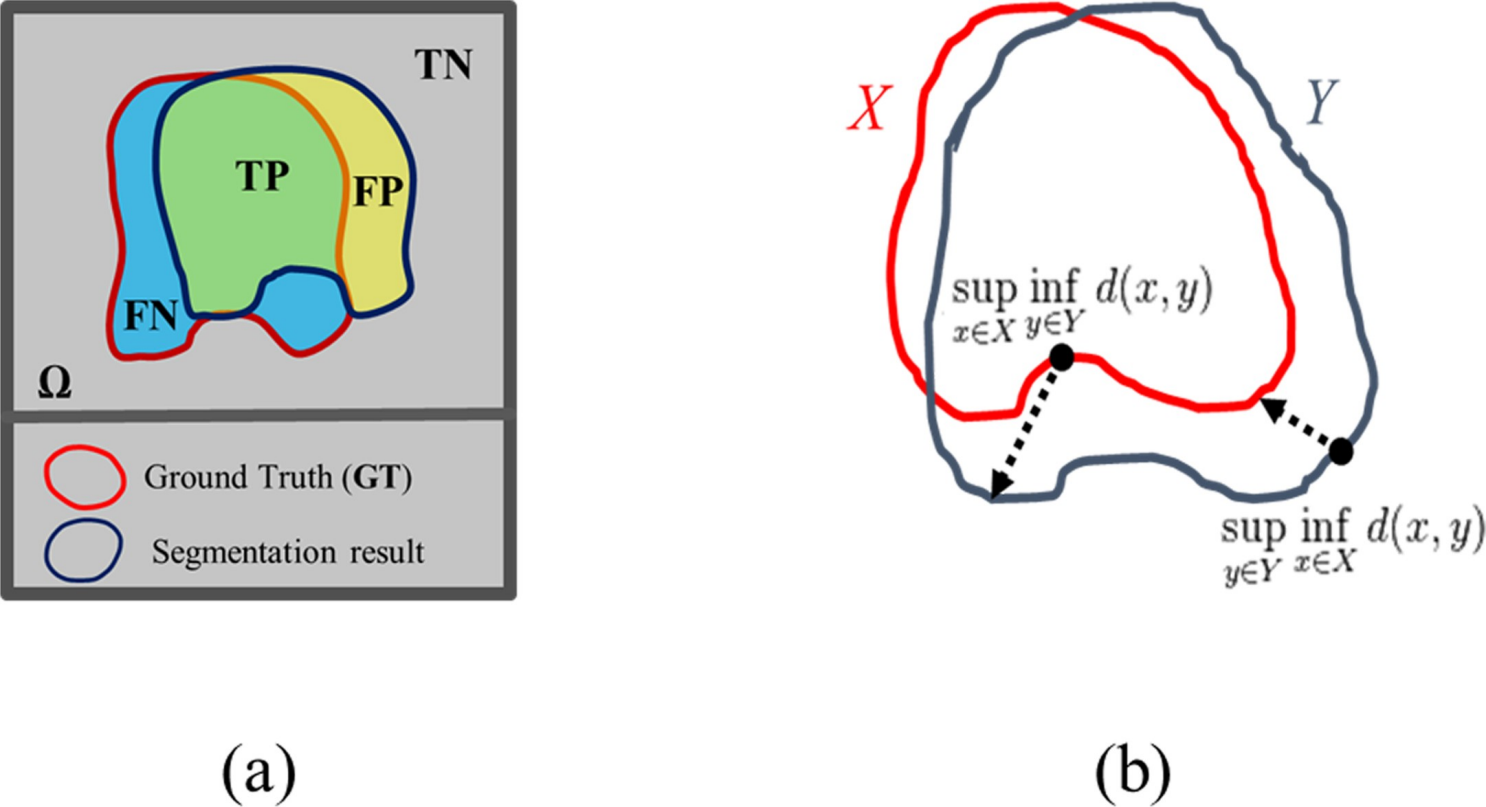
Segmentation accuracy measures. (a) In the segmentation quality measurements, there are 4 regions to be considered as: True positive (TP), false positive (FP), true negative (TN), and false negative (FN). (b) The calculation of the HD between the red line X and the blue line Y.

To evaluate the results, we calculated the segmentation accuracy compared to the ground truth (obtained from manual segmentation) using Dice similarity coefficient (DC) [38], Recall (R = $\frac{TP}{TP+FN}\;$), Precision (P = $\frac{TP}{TP+FP}\;$) and the Hausdorff distance (HD) [39]. The DC measures the concordance between two enclosed volumes as follows

$$DC=\frac{2\;TP}{FP+2TP+FN}$$ (6)

where FP represents the number of false positive (i.e. the total number of the misclassified voxels of the background), FN is the number of false negative (i.e. the total number of the misclassified voxels of the object), and TP is the true positive (i.e. total number of the correctly classified pixels), as shown in Fig 3A and 3B. On the other hand, The HD is defined as:

$$HD\left(X,Y\right)=\max\left\{sup_{x\in X}\;inf_{y\in Y}d\left(x,y\right),\;sup_{y\in Y}\;inf_{x\in X}d\left(x,y\right)\right\}$$ (7)

where X and Y are the boundaries of two different volumes. It measures how far two subsets of a metric space are from each other, as shown in Fig 3B. High DC, R, P and a low HD are desirable for good segmentation.

### Manual segmentation, ANTs, STAPLE and DeepMedic algorithms

Manual segmentation of MR images were performed by one expert operator using MANGO software (Research Imaging Institute, UTHSCSA) for determining SAT, IMAT, whole muscle, and the 3 muscle compartments considered in this study.

To compare the proposed method (**A1**) with other automatic segmentation alternatives, MRI volumes are subsequently segmented using: (**A2**) an atlas based segmentation approach using ANTs registration algorithm [40]. In this technique, to estimate accurate mapping between the same structures/tissues of each atlas subject and the test one, the nonlinear Symmetric Normalization (diffeomorphic metric mapping algorithm) has been used to obtain such a 3D deformation fields that will be applied for the corresponding labeled subject. Finally, a majority voting classifier is used to fuse the aligned atlas subjects into the final automatic segmentation. The second method (**A3**) is known as simultaneous truth and performance level estimation (STAPLE) described in [41, 42]. This algorithm considers a collection of segmentations (atlas subjects) and computes a probabilistic estimate of the final segmentation. All atlas MRI scans are aligned to the target scan and the obtained deformation fields are applied to the counterpart segmentation. The fused final segmentation is formed by estimating an optimal combination of the aligned atlas subjects incorporating a prior model for the spatial distribution of structures being segmented as well as spatial homogeneity constrains.

The total thigh MRI segmentation framework was also performed with a pre-designed CNN architecture known as DeepMedic network, which was initially designed for segmentation of brain lesions in 3-D multi-modal MRI images and won the SISS-ISLES 2015 segmentation challenge [26]. We followed the implementation steps as recommended by the developers in order to achieve the network’s best segmentation performance for segmenting SAT, IMAT, bone and three muscle compartments. To calculate the total accuracy of the DeepMedic network, we used three-fold validation method by training the network with 20 3-D MRI scans, including both SCI and ND thigh scans, and testing on the remaining 10 scans and then swapping between training and testing groups to assess the automatic segmentation results for all scans.

The manual segmentation and the proposed framework were run on a PC with 3.60 GHz, Core i7 CPU and 16.0 GB RAM. The ANTs and STAPLE algorithms were run with 3.0 GHz Core i7 Quad CPU processor and 64GB RAM. Both computers had Windows 10 OS, MATLAB R2015b and C++ programs. The CNN code was run on both 3.50 GHz, 12 Core CPU with 256.0 GB RAM and NVIDIA 1070 Ti GPU with Linux OS and Python and TensorFlow programs.

## Data availability statement

The source code for the proposed framework is available on a public GitHub repository (https://github.com/samine66m/Novel-Stochastic-Framework-for-Automatic-Segmentation-of-Human-Thigh-MRI-Volumes-and-Its-Application) and the data are available from the University of Louisville Human Subjects Protection Program Office (hsppofc@louisville.edu) for researchers who meet the criteria to access the confidential data.

## Results

### Segmentation of SAT, IMAT and bone

**Fig 4:**
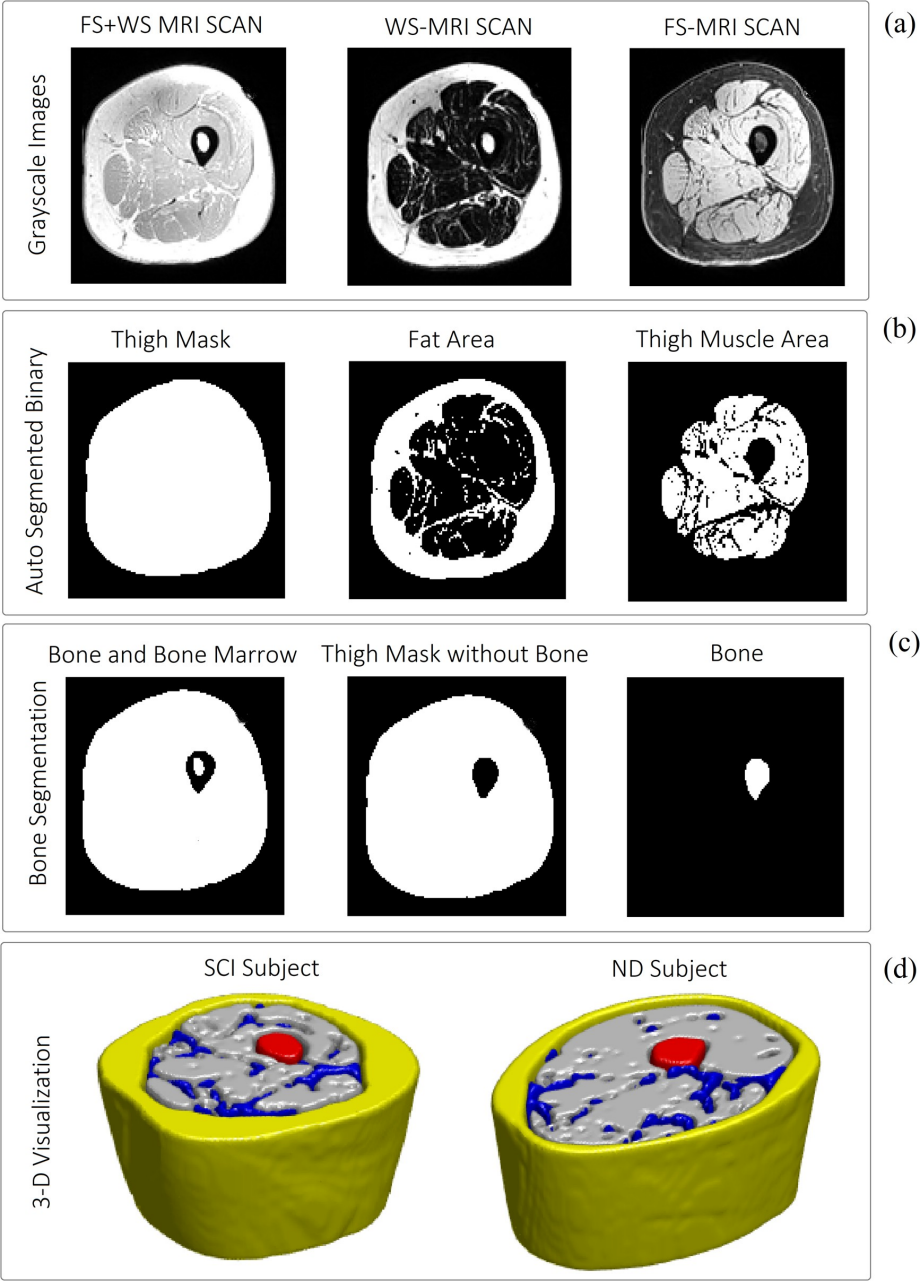
Examples for the utilization of LCDG to segment the soft tissue volumes. (a) From left to right: gray scale MR images for FS+WS, WS and FS; (b) From left to right: binary mask of total thigh area, total fat and total muscle area; (c) From left to right: steps for segmenting the bone and bone marrow; (d) 3-D representations of the segmentation results for SCI (left) and ND (right) thigh; Grey: Muscle area, Yellow: SAT, Blue: IMAT, Red: bone.

Fig 4 shows examples of the LCDG results on the sum of WS- and FS-MRI volumes, WS-MRI volumes and FS-MRI volumes (Fig 4A) for extraction of the whole thigh, whole fat, whole muscle mask (Fig 4B) as well as bone area (Fig 4C). Also, an example of 3D visualization of the final thigh segmentation results for SCI and ND is reported in Fig 4D. As explained in the methodology section, the SAT and IMAT areas were separated by using the whole muscle area as a mask on the whole fat area.

The average accuracy of the initial segmentation of fat tissue was tested by comparison of the automatic results with the manual segmentation of SAT, IMAT and thigh muscle. The comparison was made based on calculating the DC, R and P as accuracy measures. The average values of these three accuracy measures are presented in Table 1. Additionally, the individual numbers for all the accuracy metrics are presented in S2 Table.

**Table 1:** Accuracy measures for adipose tissue and total muscle area using LCDG method.

SCI				ND			
Avg. (± SD) Metrics	SAT	IMAT	Thigh Muscle	Avg. (± SD) Metrics	SAT	IMAT	Thigh Muscle
**DC**	0.97±0.03	0.96±0.05	0.99±0.01	**DC**	0.97±0.01	0.86±0.07	0.98±0.01
**P**	0.99±0.01	0.98±0.03	1.00±0.01	**P**	0.96±0.02	0.82±0.11	1.00±0.00
**R**	0.96±0.07	0.95±0.08	0.99±0.01	**R**	0.99±0.01	0.90±0.07	0.97±0.03
**HD (mm)**	3.74±3.09	11.25±4.26	4.63±2.82	**HD (mm)**	3.25±2.98	14.19±5.31	6.11±655

The average (± SD) values of accuracy measures (Dice’s coefficient (DC), Recall (R), and Precision (P) of the proposed fat segmentation approach for SCI (N = 16) and ND (N = 14) individuals.

### Muscles group segmentation

**Fig 5:**
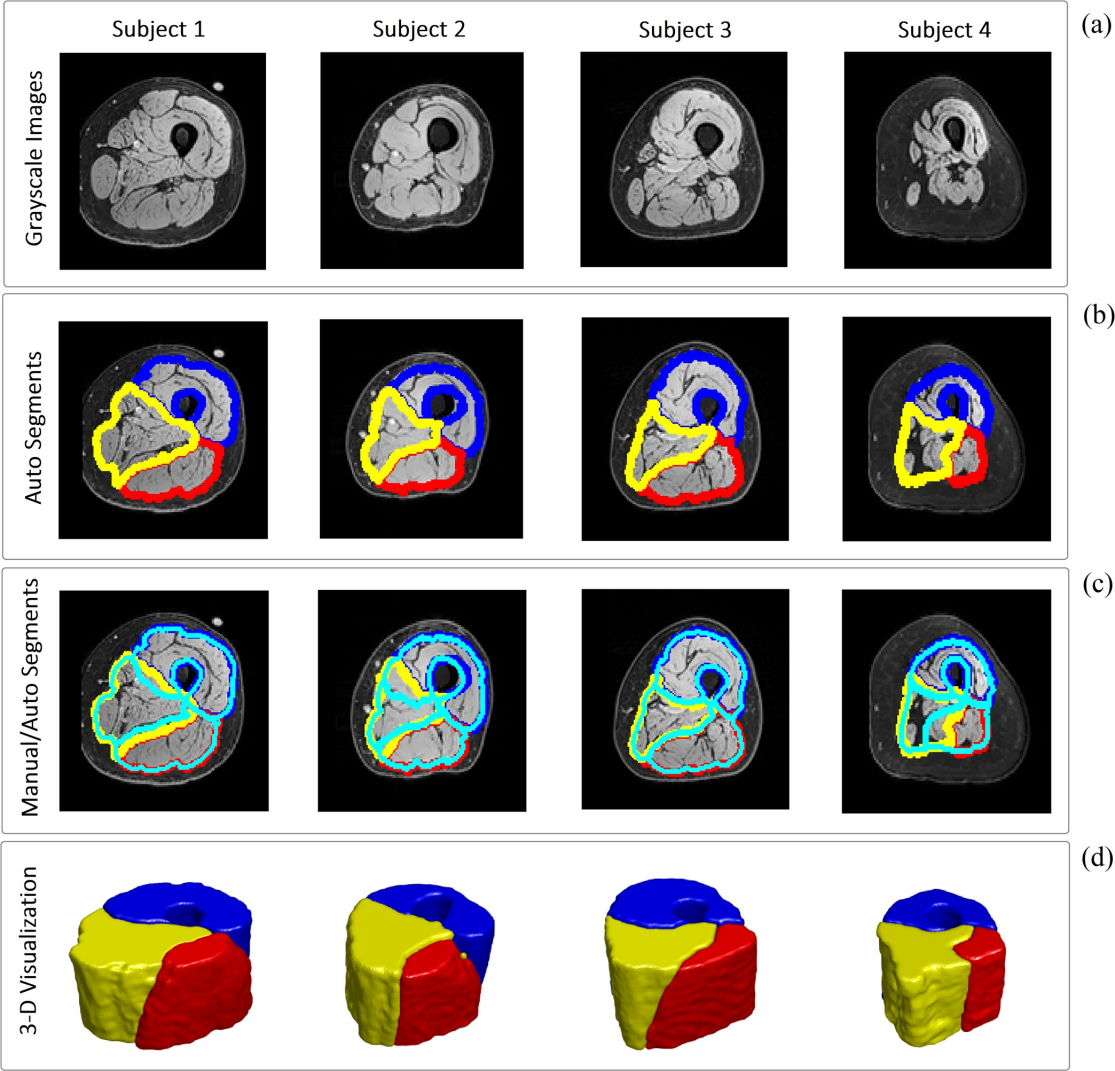
Examples of muscle group segmentation algorithm for four SCI subjects. (a) original cross sectional MR image; (b) automatic segmentation of muscle groups: blue area is extensor, red is flexor and yellow presents the medial compartment; (c) manually segmented muscle groups (cyan lines) overlaid on automatic segmentation for comparison; and (d) 3-D representation of automatic segmentation of muscle groups: blue volume is extensor, red is flexor and yellow presents the medial muscle group.

To obtain the accuracy of the three automatic muscle group segmentations, we used the common technique of “leave-one-subject-out”, where N-1 subjects are used to build the atlas and one subject was left out for testing, and we repeated this for all subjects in the SCI and ND groups separately. Fig 5 reports examples of the cross sectional area of the original grayscale MRIs (Fig 5A), the results of the automatic segmentation of the muscle groups (Fig 5B), manually segmented muscle groups overlaid on automatic segmentation (Fig 5C), and 3-D representation of automatic segmentation of muscle groups (Fig 5D).

### Comparison with ANTs and STAPLE

The average accuracy results for different methods (A1: proposed joint MGRF algorithm, A2: ANTs, and A3: STAPLE) are presented in Table 2, and related boxplot representations for DC and HD results are also reported in S3 and S4 Tables. The proposed method (A1) reaches 90.79% overall DC, 91.08% of precision, 91.19% of recall, and 16.83 mm of HD compared to DC = 84.39%, P = 90.72%, R = 81.96%, and HD = 19.64 mm for A2, and DC = 86.03%, P = 91.12%, R = 86.16%, and HD = 18.29 mm for A3. In summary, these data show that our approach leads to more accurate results compared to the other two methods that were tested in this study.

**Table 2:** Accuracy measures for segmenting three muscle compartments using the proposed method, ANTs and STAPLE.

	SCI Group				ND Group			
Method	Metric	Group 1 extensor	Group 2 flexor	Group 3 medial	Metric	Group 1 extensor	Group 2 flexor	Group 3 medial
**A1**	**DC**	0.94±0.03*	0.88±0.06*	0.89±0.05*	**DC**	0.95±0.03*	0.90±0.03*	0.89±0.06*
	**P**	0.95±0.03	0.91±0.08*	0.87±0.10	**P**	0.92±0.06	0.91±0.04*	0.89±0.07
	**R**	0.94±0.05*	0.86±0.08*	0.91±0.04*	**R**	0.97±0.03	0.90±0.07*	0.90±0.11*
	**HD(mm)**	12.98±6.44	12.84±6.96	20.47±10.2	**HD(mm)**	10.51±6.37	12.67±3.13	31.53±14.24
**A2**	**DC**	0.84±0.12	0.79±0.10	0.84±0.10	**DC**	0.89±0.08	0.85±0.09	0.85±0.09
	**P**	0.95±0.03	0.89±0.09	0.89±0.07	**P**	0.92±0.05	0.89±0.08	0.90±0.09
	**R**	0.80±0.20	0.77±0.16	0.79±0.17	**R**	0.88±0.14	0.84±0.13	0.83±0.15
	**HD(mm)**	20.22±6.07	14.75±5.90	19.80±9.29	**HD(mm)**	16.70±6.4	14.94±5.91	31.45±9.93
**A3**	**DC**	0.86±0.10	0.83±0.10	0.85±0.07	**DC**	0.89±0.08	0.87±0.07	0.86±0.07
	**P**	0.96±0.03	0.90±0.09	0.89±0.08	**P**	0.92±0.05	0.90±0.07	0.91±0.07
	**R**	0.82±0.18	0.82±0.14	0.86±0.12	**R**	0.91±0.12	0.90±0.06	0.87±0.14
	**HD(mm)**	15.60±6.71	13.42±7.27	22.41±9.48	**HD(mm)**	12.85±6.7	14.12±4.58	32.35±12.31

The average (± SD) values of accuracy measures (Dice’s coefficient (DC), Recall (R), Precision (P), and the Hausdorff distance (HD) for three methods (A1: proposed algorithm, A2: ANTs, and A3: STAPLE) for SCI and ND groups.

* Our proposed algorithm showed equal or higher accuracy than the other two methods.

### Comparison with 3-D CNN

In order to examine advantages and limitations of the deep learning approach for thigh MRI segmentation task, we have implemented the DeepMedic CNN network structure to perform the segmentation task on our entire dataset. Based on the DC similarity measure for segmentation of the three muscle groups without the IMAT, and segmentation of bone and SAT volumes, the performance of the trained CNN network was 0.93±0.03 when all 30 subjects included in the present study were considered; this value was slightly higher than that obtained using the framework proposed in this study (0.92±0.05). On the other hand, the precision measure of our framework (0.94±0.06) was slightly higher than the CNN (0.93±0.05) and the total HD calculated for our framework is only 11.82±6.72 mm which is considerably higher than the HD value calculated for the CNN network, 20.48±16.43 mm, suggesting that CNN network tended to be less accurate than the framework proposed in the present based on this accuracy measure (S5 Table).

### Comparison between SCI and ND volumes

**Fig 6:**
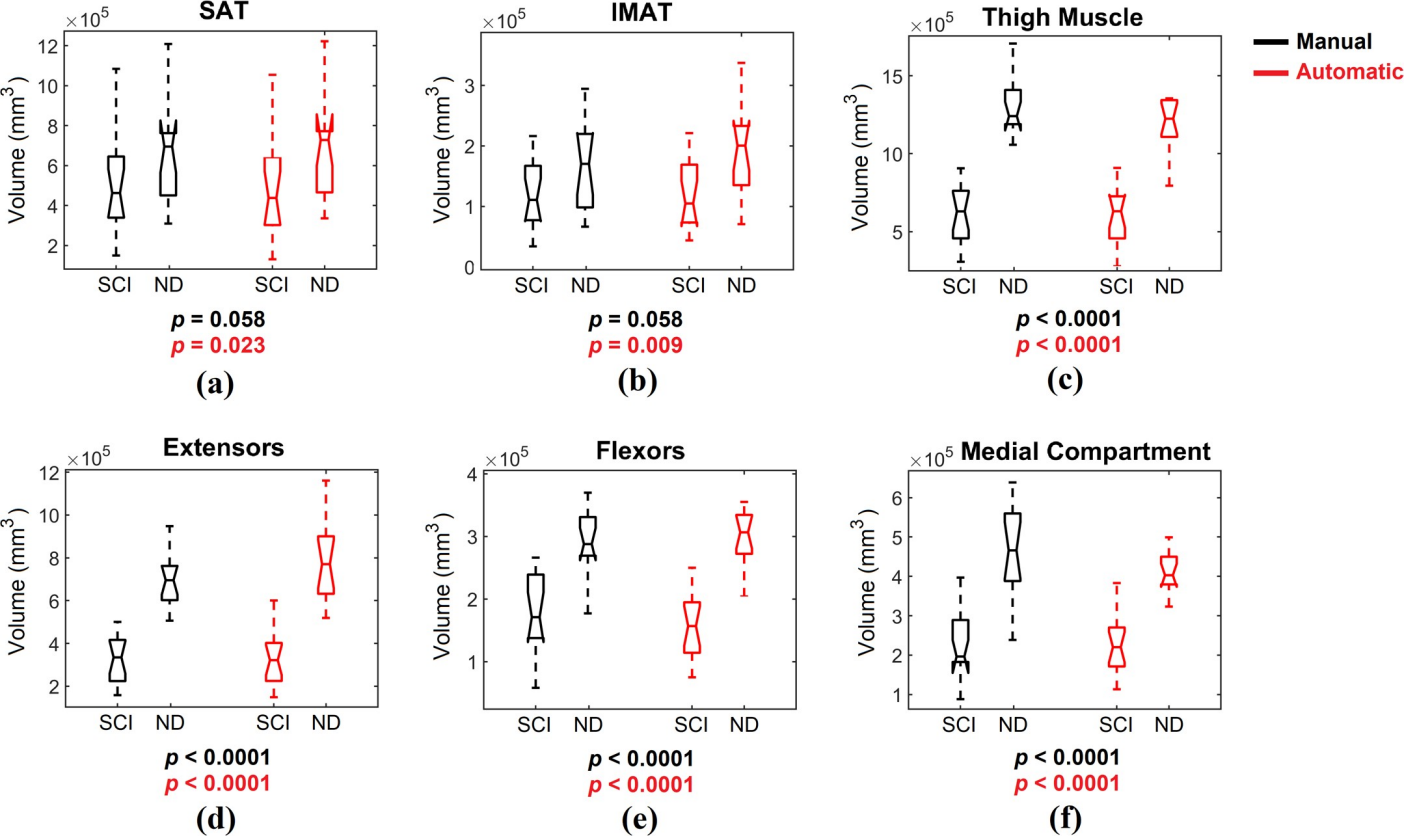
The boxplot representation of the calculated volumes and ratios for manual (black) and automatic (red) segmentation results. (a) Extensor volume; (b) Flexor volume; (c) Medial volume; (d) IMAT volume; (e) SAT volume; and (f) Total muscle volume.

The volumes of SAT, IMAT, thigh muscle, extensor muscles, flexor muscles, and medial compartment muscles were calculated based on both manual and automatic segmentation for all subjects, and presented for the SCI and ND groups separately (Fig 6). In order to determine any statistically significant difference between SCI and ND groups for each of these parameters, we used the non-parametric two-tailed Wilcoxon rank sum test with alpha level set at 0.05. This test can be used for two populations with unequal sample sizes and independent samples. The SAT and IMAT volumes were significantly greater in ND compared to SCI when the results from automatic segmentation were considered (Fig 6A and 6B), and the same trend (p = 0.058) was observed also for manual segmentation. Similarly, all muscle-related volumes were significantly greater in the ND group (p<0.0001) when both manual and automatic segmentation were considered (Fig 6C–6F). The individual numbers for this comparison are presented in S6 Table.

## Discussion

The primary focus of this study was to design, implement and examine a fully automatic framework for MRI thigh muscle and adipose tissue segmentation and volume quantification in individuals with severe SCI. The proposed segmentation framework (Fig 1) consists of three main steps: total muscle and adipose tissue segmentation based on the intensity contrast between different tissues; three muscle compartments segmentation based on three-fold stochastic integrated model (shape prior, spatial interactions and intensity); and volume quantification of the segmented parts.

In the early attempts for automatic segmentation of fat and muscle areas in MRI scans, most methods were initially designed to separate muscle and adipose tissues based on their pixel signal intensity differences. Particularly in the segmentation of MRI scans of human thighs, Barra et al. [14, 15] proposed fuzzy clustering algorithm using gray-level as voxel feature with post-processing adjustments to segment muscle and fat volumes. Histogram thresholding methods were also utilized broadly in the literature for this task [43]. Imamoglu et al. [44] also used MRI thigh scans with saliency features to extract thigh muscle volumes using morphological operations and binary fuzzy decision-based fusion. In spite of their popularity, intensity-based methods have a major setback as, for example, they cannot distinguish between different types of fat (SAT/IMAT). Therefore, the automatic segmentation methods have evolved to more advanced techniques to make these separations possible. For instance, Positano et al. [16] in 2009 added the active contour algorithm to the fuzzy clustering method to segment SAT and bone area and expectation maximization (EM) algorithm to separate IMAT from muscle area when investigating obese individuals. Similarly, Kovacs et al. [45] used the contouring method to segment fascia lata to separate SAT and IMAT in severe muscular dystrophy cases. In a recent study, Irmakci et al. [18] proposed an extended version of fuzzy connectivity method to segment the fat and whole muscle areas of thighs as well as brain and whole body tissue using multi-modal MRI images. However, segmenting the challenging task of segmenting different muscles or muscle groups in the absence of substantial intensity differences between muscles is not addressed in this study. In the present study, we also used LCDG intensity-based method for assessing thigh muscle and adipose tissue using FS and WS MRI scans of thigh. The multi-modal MRI scans of thigh muscles have been rarely used in previous automatic thigh segmentation studies. This alternative modality of MRI is particularly advantageous for thigh segmentation since it uses opposite contrasts for fat and muscle tissues in FS and WS images, and therefore by utilizing intensity-based method (LCDG) only, we could segment the whole adipose tissue in WS slices and the whole muscle area in FS images and use it as a mask to separate SAT and IMAT. Utilizing the sum of WS and FS was also used to segment the bone and bone marrow areas (Fig 4). This approach allowed us to avoid the use of a priori shape information or iterative contouring algorithm for this part of the segmentation.

The segmentation of different muscles and/or muscle groups is also a task that intensity-based segmentation methods cannot accomplish. This type of segmentation started with Andrews et al. [20], who proposed a framework for using principal component analysis (PCA)-based shape prior from the training dataset to segment the knee flexor and extensor individual muscles in MR 3-D volumes. Similarly, Baudin et al. [19, 46] introduced the iterative random walk (RW) segmentation framework for segmenting individual muscles by starting from a priori shape information and utilizing the support vector machine (SVM) method to estimate the RW parameters. A hybrid method was proposed by Andrews and Hamarneh [20] by combining the generalized log-ratio probabilistic shape model and random forest binary detector to segment each individual muscle of the human thigh. Utilizing the atlas-based methods has been gained more attention in recent years with Ahmad et al. [21] framework of atlas construction and image registration to segment the quadriceps muscle group. Another atlas-based segmentation method was proposed by Troter et al. [22] to segment four individual muscle volumes inside the quadriceps group by using semi-automated single-atlas and fully automated multiple-atlas approaches and suggesting that the single-atlas method was more robust for individual muscle segmentation and has a better accuracy. In 2017, Orgier et al. [47] have proposed a new semi-automatic technique for segmenting the same four quadriceps muscles by manually segmenting the top and bottom slices and using the propagating non-linear registration approach to segment the middle slices. In the thigh volumes segmentation application, we were particularly interested in segmenting knee extensors and knee flexors muscle groups because of their important functional role in human movement generation. Unlike previous studies that only used prior shape information for muscle segmentation, our proposed method uses all three components of shape, spatial (MGRF) and intensity (LCDG) to determine if a given voxel belongs to any of the three muscle groups considered (knee extensors, knee flexors, or medial compartment) (Fig 5). The segmentation method proposed in this study was applied on a group of individuals with severe SCI and on a group of ND individuals. In order to build a generic thigh segmentation atlas for ND group, we recruited individuals considered as normal (N = 3), overweight (N = 5) and obese (N = 6) as for their BMI [48]. The DC accuracy values (Table 1) suggest that the fat and thigh muscle area, which were segmented using the LCDG intensity-based approach showed overall high accuracy values, which were equal to 90.97 ± 6.74% for the SCI group and 93.91 ± 7.02% for the ND group. For the muscle compartment segmentation, the proposed method showed on average 90.41 ± 5.59% accuracy for SCI group and 91.18 ± 5.02% for ND (breakdown numbers are presented in Table 2). The very similar accuracy for the muscle compartment segmentation between SCI and ND is noteworthy, seen as the SCI group showed substantial inter-individual differences, as exemplified in Fig 5 (see subject 4 compared to the other three subjects).

In order to further evaluate our segmentation method, we also compared the accuracy results related to muscle compartment segmentation with those obtained from two other well-known segmentation methods (ANTs and STAPLE). The three-fold integrated model proposed in this study showed an overall greater accuracy compared to ANTs and STAPLE, as for the accuracy measures that were calculated (DC, R, P and HD; Table 2). This may be due to the positive effect of integrating both the appearance and spatial models with the prior shape information from the atlas into a three joint MGRF model. In particular, the prior atlas enables the proposed approach to use known muscle anatomy to distinguish and correctly classify different muscle compartments that have the same appearance, while the spatial models handle any inhomogeneity that may exist within a muscle compartment.

We also performed the entire thigh muscle and fat segmentation task with the well-known DeepMedic 3-D CNN structure to compare its performance with the stochastic-based algorithm proposed in this study. The different trends observed for DC and HD indexes may be due to the fact that the falsely segmented voxels of the CNN method were mostly happened far from the boundary of the targeted areas which have longer distances from the actual borders (greater HD) whereas for the joint MGRF method, the falsely segmented voxels mostly happened near the borders of the muscle groups which lead to smaller HD values. While the CNN performance was comparable to the proposed method for muscle MRI segmentation task, implementing the CNN code required extensive memory and computations as well as expertise in programming in Linux OS, Python and TensorFlow. The total executive run-time for segmenting the entire database using 3-D CNN was 52.0 hours on GPU while the total run-time for the proposed method was only 5.3 hours on CPU (S7 Table). We also attempted to run the DeepMedic software multiple times over 4 weeks for the training step using a regular CPU, but we were unable to properly train the network. The CNN training duration is still dramatically longer than the processing time of the proposed framework. However, once the CNN algorithm is trained, the processing time for segmenting the testing subjects was relatively low. We have shown that the 3-D CNN-based method can be quickly adjusted to thigh MRI segmentation task without any changes to the network structure. Also the CNN can be trained on part of the dataset and segment the other part (test scans) with acceptable accuracy and relatively fast using GPU; however, as the number of test subjects grows over time in a clinical setting, it would be desirable to re-train the network or use transfer learning [49] to improve the segmentation accuracy. Conversely, in the proposed framework, all the previously segmented and reviewed scans can be utilized in the future atlases to guide the segmentation of a new thigh MRI scan without substantial additional computational cost.

Finally, we compared the volumes of SAT, IMAT, thigh muscle, knee extensors, knee flexors, and medial compartment between SCI and ND groups using the results obtained from both automatic and manual segmentation (Fig 6). The main goal of this comparison was to examine whether the same conclusion in terms of physiological differences between the two groups could be achieved using both segmentation methods. SAT and IMAT volumes were significantly greater (p = 0.023 and p = 0.009, respectively) in ND using automatic segmentation outcomes; a similar trend (p = 0.058) was also observed using the volumes calculated from manual segmentation (Fig 6A and 6B). The greater adipose tissue volumes found in ND is an unexpected finding, as most of the data reported in the literature show that after SCI there is an increase in body fat mass as well as IMAT in the thigh [6, 50]. The ratio between IMAT and thigh muscle volume calculated in the SCI group of the present study (19.8%) is within the range observed in other SCI individuals [6, 50]. On the other hand, most of the ND individuals enrolled in the present study were either overweight or obese, and these conditions can result in increased SAT and IMAT volumes [51].

Thigh muscle volume and the volumes of the three investigated muscle compartments were significantly greater in ND individuals compared to the SCI group when outcomes from both automatic and manual segmentation were considered (Fig 6C–6F).These findings are in agreements with previous studies that showed marked SCI-induced muscle atrophy [5, 6].

In conclusion, we developed a novel and accurate MRI-based segmentation framework that can automatically segment thigh subcutaneous and intermuscular adipose tissue as well as muscle tissue related to knee extensors and knee flexors in individuals with SCI. These parameters have important health and functional implications in the SCI population and the proposed segmentation method can facilitate the use of MRI to assess individual characteristics and possibly the effects of different interventions. This framework could be further improved by increasing the MRI resolution, which would allow an accurate segmentation of intra-muscular adipose tissue and individual muscles, and by increasing the number of thigh MRI slices from the 50 central to the whole thigh in order to make a more comprehensive assessment of the different volumes.

## Supporting information

S1 Table Clinical characteristics. Clinical characteristics of research participants.
(DOCX)

S2 Table Accuracy values of proposed method. Accuracy values for each segmented volume including Extensor muscle group, Flexor muscle group, Medial muscle group, IMAT, SAT, Muscle Area, based on Dice similarity index (SI), Precision (P), Recall (R) and Hausdorff distance (HD) measures.
(DOCX)

S3 Table Accuracy values of ANTs method. Accuracy values for segmenting extensor, flexor and medial muscle compartments based on Dice similarity index (SI), Precision (P), Recall (R) and Hausdorff distance (HD) measures.
(DOCX)

S4 Table Accuracy values of STAPLE method. Accuracy values for segmenting extensor, flexor and medial muscle compartments based on Dice similarity index (SI), Precision (P), Recall (R) and Hausdorff distance (HD) measures.
(DOCX)

S5 Table Accuracy values of 3D CNN-based segmentation method and the proposed method. Accuracy values for segmenting SAT, Extensor compartment without IMAT, Flexor compartment without IMAT and Medial compartment based on Dice similarity index (SI), Precision (P), Recall (R) and Hausdorff distance (HD) measures.
(DOCX)

S6 Table Comparison of automatic and manual segmentation. Calculated volumes and ratios for manual and automatic segmentation results for Extensor volume, Flexor volume, Medial volume, IMAT volume, SAT volume and Total muscle volume.
(DOCX)

S7 Table Execution run-time. Run-time values for the proposed segmentation framework on CPU and 3D CNN method on GPU.
(DOCX)
