# Correction: Novel stochastic framework for automatic segmentation of human thigh MRI volumes and its applications in spinal cord injured individuals

**DOI:** 10.1371/journal.pone.0219810

**Published:** 2019-07-16

**Authors:** Samineh Mesbah, Ahmed M. Shalaby, Sean Stills, Ahmed Soliman, Andrea Willhite, Susan J. Harkema, Enrico Rejc, Ayman S. El-Baz

The fourth author’s name is incorrect. The correct name is: Ahmed Soliman. The correct citation is: Mesbah S, Shalaby AM, Stills S, Soliman A, Willhite A, Harkema SJ, et al. (2019) Novel stochastic framework for automatic segmentation of human thigh MRI volumes and its applications in spinal cord injured individuals. PLoS ONE 14(5): e0216487. https://doi.org/10.1371/journal.pone.0216487.
