# Supplementary material for: Novel stochastic framework for automatic segmentation of human thigh MRI volumes and its applications in spinal cord injured individuals
Source: PLoS One. 2019 May 9;14(5):e0216487. doi: 10.1371/journal.pone.0216487 (PMC6508923; doi:10.1371/journal.pone.0216487)
Supplement: S1 Table — Clinical characteristics of research participants. (DOCX) [file pone.0216487.s001.docx]

**S1 Table.** **Clinical characteristics.**  Clinical characteristics of research participants.

| **SCI ID** | **Age (Yrs)** | **TSI (Yrs)** | **Stature (m)** | **Body Mass (Kg)** | **BMI** | **Gender** | **AIS** | **ND ID** | **Age (Yrs)** | **Stature (m)** | **Body Mass (Kg)** | **BMI** | **Gender** | **Condition** |
| --- | --- | --- | --- | --- | --- | --- | --- | --- | --- | --- | --- | --- | --- | --- |
| Subject 01 | 23.9 | 7.1 | 1.85 | 90.7 | 26.4 | M | A | Subject 01 | 35.2 | 1.88 | 95.3 | 27.0 | M | OW |
| Subject 02 | 26.2 | 5.1 | 1.80 | 63.5 | 19.5 | M | A | Subject 02 | 27.5 | 1.75 | 111.1 | 36.2 | M | OB |
| Subject 03 | 39.4 | 9.8 | 1.73 | 77.1 | 25.8 | M | A | Subject 03 | 31.8 | 1.85 | 99.8 | 29.0 | M | OW |
| Subject 04 | 20.8 | 3.0 | 1.73 | 68.0 | 22.8 | F | B | Subject 04 | 27.6 | 1.78 | 99.8 | 31.6 | M | OB |
| Subject 05 | 50.4 | 33.3 | 1.88 | 79.4 | 22.5 | M | A | Subject 05 | 27.5 | 1.73 | 72.6 | 24.3 | M | H |
| Subject 06 | 25.6 | 1.9 | 1.73 | 81.6 | 27.4 | M | B | Subject 06 | 28.0 | 1.83 | 104.3 | 31.2 | M | OB |
| Subject 07 | 34.9 | 3.7 | 1.78 | 61.2 | 19.4 | M | A | Subject 07 | 36.3 | 1.75 | 102.1 | 33.2 | M | OB |
| Subject 08 | 39.9 | 1.6 | 1.80 | 83.0 | 25.5 | M | B | Subject 08 | 27.4 | 1.93 | 122.5 | 32.9 | M | OB |
| Subject 09 | 22.7 | 2.6 | 1.88 | 81.6 | 23.1 | M | A | Subject 09 | 29.6 | 1.70 | 87.1 | 30.1 | F | OB |
| Subject 10 | 48.5 | 2.0 | 1.83 | 90.7 | 27.1 | M | A | Subject 10 | 22.7 | 1.80 | 81.6 | 25.1 | M | OW |
| Subject 11 | 35.2 | 9.8 | 1.80 | 81.6 | 25.1 | M | C | Subject 11 | 24.5 | 1.70 | 77.1 | 26.6 | F | OW |
| Subject 12 | 25.2 | 9.1 | 1.88 | 65.8 | 18.6 | M | B | Subject 12 | 25.8 | 1.73 | 83.9 | 28.1 | M | OW |
| Subject 13 | 34.4 | 5.2 | 1.88 | 86.2 | 24.4 | M | A | Subject 13 | 25.6 | 2.08 | 90.7 | 20.9 | M | H |
| Subject 14 | 28.2 | 10.0 | 1.60 | 86.2 | 33.7 | F | A | Subject 14 | 29.0 | 1.70 | 68.0 | 23.5 | F | H |
| Subject 15 | 34.9 | 0.9 | 1.78 | 90.7 | 28.7 | M | A | **AVERAGE** | 28.5 | 1.80 | 92.6 | 28.5 |  |  |
| Subject 16 | 22.2 | 2.6 | 1.55 | 47.6 | 19.8 | F | B | **SD** | 3.8 | 0.11 | 15.3 | 4.3 |  |  |
| **AVERAGE** | 32.0 | 6.7 | 1.78 | 77.2 | 24.4 |  |  |  |  |  |  |  |  |  |
| **SD** | 9.2 | 7.8 | 0.10 | 12.5 | 4.0 |  |  |  |  |  |  |  |  |  |

AIS: American Spinal Injury Association (ASIA) Impairment Scale

TSI: Time since injury

BMI: Body Mass Index

OW: Over Weight

OB: Obese

H: Healthy

SCI: Spinal Cord Injury

ND: Non-disabled
