# Supplementary material for: Novel stochastic framework for automatic segmentation of human thigh MRI volumes and its applications in spinal cord injured individuals
Source: PLoS One. 2019 May 9;14(5):e0216487. doi: 10.1371/journal.pone.0216487 (PMC6508923; doi:10.1371/journal.pone.0216487)
Supplement: S2 Table — Accuracy values for each segmented volume including Extensor muscle group, Flexor muscle group, Medial muscle group, IMAT, SAT, Muscle Area, based on Dice similarity index (SI), Precision (P), Recall (R) and Hausdorff distance (HD) measures. (DOCX) [file pone.0216487.s002.docx]

**S2 Table.** **Accuracy values of proposed method**. Accuracy values for each segmented volume including Extensor muscle group, Flexor muscle group, Medial muscle group, IMAT, SAT, Muscle Area, based on Dice similarity index (SI), Precision (P), Recall (R) and Hausdorff distance (HD) measures.

| **SI (Dice)** | **SCI ID** | **EXTENSOR** | **FLEXOR** | **MEDIAL** | **IMAT** | **SAT** | **MUSCLE** | **ND ID** | **EXTENSOR** | **FLEXOR** | **MEDIAL** | **IMAT** | **SAT** | **MUSCLE** |
| --- | --- | --- | --- | --- | --- | --- | --- | --- | --- | --- | --- | --- | --- | --- |
|  | Subject 01 | 0.97 | 0.95 | 0.94 | 0.98 | 0.99 | 1.00 | Subject 01 | 0.97 | 0.94 | 0.94 | 0.83 | 0.96 | 0.98 |
|  | Subject 02 | 0.97 | 0.93 | 0.93 | 0.97 | 0.99 | 1.00 | Subject 02 | 0.96 | 0.92 | 0.92 | 0.84 | 0.96 | 0.97 |
|  | Subject 03 | 0.97 | 0.92 | 0.95 | 0.92 | 0.98 | 0.98 | Subject 03 | 0.93 | 0.90 | 0.88 | 0.77 | 0.97 | 0.97 |
|  | Subject 04 | 0.93 | 0.93 | 0.89 | 1.00 | 0.99 | 1.00 | Subject 04 | 0.89 | 0.87 | 0.77 | 0.93 | 0.99 | 1.00 |
|  | Subject 05 | 0.97 | 0.93 | 0.89 | 0.99 | 1.00 | 1.00 | Subject 05 | 0.97 | 0.92 | 0.94 | 0.85 | 0.95 | 0.97 |
|  | Subject 06 | 0.94 | 0.94 | 0.93 | 0.93 | 0.98 | 0.99 | Subject 06 | 0.96 | 0.83 | 0.88 | 0.79 | 0.98 | 0.96 |
|  | Subject 07 | 0.94 | 0.80 | 0.76 | 0.98 | 0.99 | 1.00 | Subject 07 | 0.88 | 0.89 | 0.78 | 0.95 | 0.99 | 1.00 |
|  | Subject 08 | 0.95 | 0.90 | 0.89 | 0.97 | 0.90 | 0.99 | Subject 08 | 0.95 | 0.93 | 0.89 | 0.79 | 0.98 | 0.97 |
|  | Subject 09 | 0.96 | 0.85 | 0.81 | 0.82 | 0.91 | 0.98 | Subject 09 | 0.97 | 0.93 | 0.95 | 0.86 | 0.98 | 0.97 |
|  | Subject 10 | 0.93 | 0.91 | 0.82 | 0.97 | 0.91 | 1.00 | Subject 10 | 0.97 | 0.94 | 0.96 | 0.94 | 0.98 | 1.00 |
|  | Subject 11 | 0.96 | 0.95 | 0.95 | 0.94 | 0.96 | 1.00 | Subject 11 | 0.96 | 0.94 | 0.95 | 0.95 | 0.98 | 1.00 |
|  | Subject 12 | 0.94 | 0.88 | 0.89 | 1.00 | 0.98 | 1.00 | Subject 12 | 0.97 | 0.87 | 0.91 | 0.92 | 0.97 | 1.00 |
|  | Subject 13 | 0.94 | 0.84 | 0.89 | 0.98 | 0.99 | 1.00 | Subject 13 | 0.93 | 0.87 | 0.81 | 0.73 | 0.97 | 0.98 |
|  | Subject 14 | 0.92 | 0.85 | 0.89 | 1.00 | 1.00 | 1.00 | Subject 14 | 0.93 | 0.87 | 0.89 | 0.88 | 0.98 | 0.99 |
|  | Subject 15 | 0.87 | 0.72 | 0.87 | 0.98 | 1.00 | 1.00 | **SD** | 0.03 | 0.03 | 0.06 | 0.07 | 0.01 | 0.01 |
|  | Subject 16 | 0.93 | 0.84 | 0.90 | 1.00 | 1.00 | 1.00 | **Averge** | 0.95 | 0.90 | 0.89 | 0.86 | 0.97 | 0.98 |
|  | **SD** | 0.03 | 0.06 | 0.05 | 0.05 | 0.03 | 0.01 | **Average ND** | 0.93 | 0.06 |  |  |  |  |
|  | **Averge** | 0.94 | 0.88 | 0.89 | 0.96 | 0.97 | 0.99 |  |  |  |  |  |  |  |
|  | **Average SCI** | 0.94 | 0.06 |  |  |  |  |  |  |  |  |  |  |  |
|  | **SCI+ND Average** | 0.93 | 0.06 |  |  |  |  |  |  |  |  |  |  |  |
| **Percision (P)** | **SCI ID** | **EXTENSOR** | **FLEXOR** | **MEDIAL** | **IMAT** | **SAT** | **MUSCLE** | **ND ID** | **EXTENSOR** | **FLEXOR** | **MEDIAL** | **IMAT** | **SAT** | **MUSCLE** |
|  | Subject 01 | 0.96 | 0.95 | 0.95 | 1.00 | 0.99 | 1.00 | Subject 01 | 0.98 | 0.95 | 0.93 | 0.79 | 0.95 | 0.99 |
|  | Subject 02 | 0.97 | 0.97 | 0.92 | 1.00 | 1.00 | 1.00 | Subject 02 | 0.94 | 0.87 | 0.96 | 0.79 | 0.93 | 1.00 |
|  | Subject 03 | 0.97 | 0.96 | 0.95 | 0.91 | 0.96 | 1.00 | Subject 03 | 0.87 | 0.86 | 0.95 | 0.68 | 0.97 | 1.00 |
|  | Subject 04 | 0.96 | 0.96 | 0.87 | 1.00 | 1.00 | 1.00 | Subject 04 | 0.81 | 0.93 | 0.88 | 0.92 | 0.99 | 1.00 |
|  | Subject 05 | 0.97 | 0.99 | 0.86 | 1.00 | 1.00 | 1.00 | Subject 05 | 0.95 | 0.93 | 0.90 | 0.77 | 0.91 | 1.00 |
|  | Subject 06 | 0.93 | 0.93 | 0.95 | 1.00 | 0.97 | 0.99 | Subject 06 | 0.97 | 0.92 | 0.82 | 0.68 | 0.97 | 1.00 |
|  | Subject 07 | 0.96 | 0.91 | 0.68 | 0.98 | 0.99 | 1.00 | Subject 07 | 0.78 | 0.85 | 0.96 | 0.95 | 0.99 | 1.00 |
|  | Subject 08 | 0.95 | 0.86 | 0.91 | 1.00 | 1.00 | 0.99 | Subject 08 | 0.98 | 0.94 | 0.82 | 0.69 | 0.98 | 1.00 |
|  | Subject 09 | 0.98 | 0.98 | 0.69 | 0.99 | 1.00 | 0.96 | Subject 09 | 0.96 | 0.92 | 0.94 | 0.78 | 0.97 | 1.00 |
|  | Subject 10 | 1.00 | 0.97 | 0.71 | 0.93 | 1.00 | 1.00 | Subject 10 | 0.96 | 0.92 | 0.94 | 0.95 | 0.98 | 1.00 |
|  | Subject 11 | 0.95 | 0.98 | 0.96 | 1.00 | 0.96 | 1.00 | Subject 11 | 0.94 | 0.93 | 0.93 | 0.95 | 0.98 | 1.00 |
|  | Subject 12 | 0.94 | 0.85 | 0.93 | 0.99 | 1.00 | 1.00 | Subject 12 | 0.95 | 0.97 | 0.84 | 0.93 | 0.96 | 1.00 |
|  | Subject 13 | 0.92 | 0.90 | 0.91 | 0.97 | 0.99 | 1.00 | Subject 13 | 0.93 | 0.92 | 0.70 | 0.75 | 0.96 | 0.99 |
|  | Subject 14 | 0.89 | 0.91 | 0.97 | 1.00 | 1.00 | 1.00 | Subject 14 | 0.87 | 0.81 | 0.96 | 0.88 | 0.97 | 1.00 |
|  | Subject 15 | 0.97 | 0.72 | 0.80 | 0.98 | 1.00 | 1.00 | **SD** | 0.06 | 0.04 | 0.07 | 0.11 | 0.02 | 0.00 |
|  | Subject 16 | 0.90 | 0.80 | 0.93 | 0.99 | 1.00 | 1.00 | **Averge** | 0.92 | 0.91 | 0.89 | 0.82 | 0.96 | 1.00 |
|  | **SD** | 0.03 | 0.08 | 0.10 | 0.03 | 0.01 | 0.01 | **Average ND** | 0.92 | 0.08 |  |  |  |  |
|  | **Averge** | 0.95 | 0.91 | 0.87 | 0.98 | 0.99 | 1.00 |  |  |  |  |  |  |  |
|  | **Average SCI** | 0.95 | 0.07 |  |  |  |  |  |  |  |  |  |  |  |
|  | **SCI+ND Average** | 0.93 | 0.08 |  |  |  |  |  |  |  |  |  |  |  |
| **Recall (R)** | **SCI ID** | **EXTENSOR** | **FLEXOR** | **MEDIAL** | **IMAT** | **SAT** | **MUSCLE** | **ND ID** | **EXTENSOR** | **FLEXOR** | **MEDIAL** | **IMAT** | **SAT** | **MUSCLE** |
|  | Subject 01 | 0.98 | 0.94 | 0.92 | 0.97 | 1.00 | 1.00 | Subject 01 | 0.96 | 0.93 | 0.96 | 0.89 | 0.98 | 0.97 |
|  | Subject 02 | 0.97 | 0.89 | 0.94 | 0.95 | 0.99 | 1.00 | Subject 02 | 0.98 | 0.97 | 0.89 | 0.91 | 0.99 | 0.95 |
|  | Subject 03 | 0.97 | 0.88 | 0.94 | 0.93 | 1.00 | 0.96 | Subject 03 | 0.99 | 0.95 | 0.81 | 0.89 | 0.97 | 0.95 |
|  | Subject 04 | 0.90 | 0.89 | 0.90 | 1.00 | 0.99 | 1.00 | Subject 04 | 1.00 | 0.82 | 0.68 | 0.95 | 1.00 | 1.00 |
|  | Subject 05 | 0.97 | 0.88 | 0.93 | 0.99 | 1.00 | 1.00 | Subject 05 | 0.98 | 0.91 | 0.97 | 0.95 | 0.99 | 0.94 |
|  | Subject 06 | 0.95 | 0.96 | 0.90 | 0.88 | 1.00 | 1.00 | Subject 06 | 0.95 | 0.76 | 0.96 | 0.96 | 0.98 | 0.93 |
|  | Subject 07 | 0.92 | 0.71 | 0.87 | 0.98 | 0.99 | 1.00 | Subject 07 | 1.00 | 0.93 | 0.65 | 0.96 | 0.99 | 1.00 |
|  | Subject 08 | 0.96 | 0.94 | 0.87 | 0.94 | 0.82 | 0.99 | Subject 08 | 0.91 | 0.92 | 0.96 | 0.95 | 0.99 | 0.94 |
|  | Subject 09 | 0.94 | 0.74 | 0.97 | 0.70 | 0.83 | 1.00 | Subject 09 | 0.98 | 0.93 | 0.96 | 0.96 | 0.99 | 0.94 |
|  | Subject 10 | 0.87 | 0.85 | 0.97 | 1.00 | 0.84 | 1.00 | Subject 10 | 0.98 | 0.96 | 0.97 | 0.81 | 1.00 | 0.96 |
|  | Subject 11 | 0.97 | 0.93 | 0.94 | 0.90 | 0.96 | 1.00 | Subject 11 | 0.98 | 0.95 | 0.96 | 0.94 | 0.99 | 1.00 |
|  | Subject 12 | 0.94 | 0.90 | 0.85 | 1.00 | 0.97 | 1.00 | Subject 12 | 0.99 | 0.80 | 0.99 | 0.91 | 0.97 | 1.00 |
|  | Subject 13 | 0.96 | 0.79 | 0.87 | 0.98 | 0.99 | 1.00 | Subject 13 | 0.92 | 0.82 | 0.95 | 0.72 | 0.98 | 0.98 |
|  | Subject 14 | 0.95 | 0.79 | 0.83 | 1.00 | 0.99 | 1.00 | Subject 14 | 0.99 | 0.95 | 0.83 | 0.89 | 0.99 | 0.99 |
|  | Subject 15 | 0.78 | 0.73 | 0.95 | 0.99 | 1.00 | 1.00 | **SD** | 0.03 | 0.07 | 0.11 | 0.07 | 0.01 | 0.03 |
|  | Subject 16 | 0.96 | 0.88 | 0.88 | 1.00 | 1.00 | 1.00 | **Averge** | 0.97 | 0.90 | 0.90 | 0.90 | 0.99 | 0.97 |
|  | **SD** | 0.05 | 0.08 | 0.04 | 0.08 | 0.07 | 0.01 | **Average ND** | 0.94 | 0.07 |  |  |  |  |
|  | **Averge** | 0.94 | 0.86 | 0.91 | 0.95 | 0.96 | 0.99 |  |  |  |  |  |  |  |
|  | **Average SCI** | 0.93 | 0.07 |  |  |  |  |  |  |  |  |  |  |  |
|  | **SCI+ND Average** | 0.94 | 0.07 |  |  |  |  |  |  |  |  |  |  |  |
| **HD** | **SCI ID** | **EXTENSOR** | **FLEXOR** | **MEDIAL** | **IMAT** | **SAT** | **MUSCLE** | **ND ID** | **EXTENSOR** | **FLEXOR** | **MEDIAL** | **IMAT** | **SAT** | **MUSCLE** |
|  | Subject 01 | 9.49 | 10.05 | 41.88 | 10.05 | 2.45 | 5.39 | Subject 01 | 6.16 | 10.49 | 36.36 | 12.69 | 1.73 | 3.61 |
|  | Subject 02 | 8.66 | 10.30 | 18.97 | 11.45 | 3.46 | 4.47 | Subject 02 | 9.27 | 14.87 | 32.59 | 10.68 | 13.34 | 8.00 |
|  | Subject 03 | 22.23 | 8.83 | 17.09 | 14.04 | 4.47 | 11.40 | Subject 03 | 14.04 | 16.58 | 20.35 | 12.21 | 2.24 | 5.10 |
|  | Subject 04 | 7.48 | 5.74 | 17.52 | 4.47 | 2.00 | 2.24 | Subject 04 | 23.11 | 11.05 | 28.62 | 17.75 | 3.00 | 5.00 |
|  | Subject 05 | 10.05 | 7.55 | 8.60 | 10.00 | 1.41 | 2.45 | Subject 05 | 4.12 | 7.35 | 26.08 | 9.27 | 2.00 | 5.00 |
|  | Subject 06 | 13.15 | 8.77 | 14.28 | 11.31 | 3.00 | 4.00 | Subject 06 | 7.14 | 15.30 | 17.89 | 27.77 | 3.61 | 27.86 |
|  | Subject 07 | 4.90 | 16.52 | 20.15 | 11.31 | 1.41 | 2.83 | Subject 07 | 21.77 | 11.00 | 27.28 | 15.36 | 3.00 | 5.74 |
|  | Subject 08 | 10.25 | 14.59 | 15.26 | 10.49 | 11.75 | 5.10 | Subject 08 | 13.08 | 13.19 | 53.61 | 9.85 | 2.24 | 6.71 |
|  | Subject 09 | 8.25 | 16.76 | 17.49 | 17.26 | 2.24 | 3.32 | Subject 09 | 4.12 | 12.00 | 25.98 | 8.31 | 3.00 | 3.16 |
|  | Subject 10 | 12.08 | 13.64 | 20.22 | 15.52 | 2.00 | 2.45 | Subject 10 | 4.47 | 9.64 | 40.91 | 21.02 | 1.41 | 3.32 |
|  | Subject 11 | 7.00 | 5.83 | 7.35 | 12.88 | 2.83 | 1.00 | Subject 11 | 5.39 | 8.54 | 5.39 | 16.31 | 3.00 | 5.39 |
|  | Subject 12 | 22.02 | 10.30 | 9.22 | 5.74 | 4.58 | 2.24 | Subject 12 | 6.40 | 14.14 | 59.51 | 9.68 | 3.24 | 0.00 |
|  | Subject 13 | 28.32 | 19.24 | 25.08 | 18.44 | 10.82 | 8.31 | Subject 13 | 15.65 | 15.75 | 42.54 | 13.60 | 1.73 | 3.61 |
|  | Subject 14 | 15.36 | 11.36 | 40.80 | 7.87 | 2.24 | 5.83 | Subject 14 | 12.37 | 17.46 | 24.35 | 14.14 | 2.00 | 3.00 |
|  | Subject 15 | 16.40 | 34.50 | 21.21 | 15.00 | 2.24 | 8.83 | **SD** | 6.37 | 3.13 | 14.24 | 5.31 | 2.98 | 6.55 |
|  | Subject 16 | 12.08 | 11.40 | 32.39 | 4.12 | 3.00 | 4.24 | **Averge** | 10.51 | 12.67 | 31.53 | 14.19 | 3.25 | 6.11 |
|  | **SD** | 6.44 | 6.96 | 10.24 | 4.26 | 3.09 | 2.82 | **Average ND** | 13.04 | 11.64 |  |  |  |  |
|  | **Averge** | 12.98 | 12.84 | 20.47 | 11.25 | 3.74 | 4.63 |  |  |  |  |  |  |  |
|  | **Average SCI** | 10.99 | 8.27 |  |  |  |  |  |  |  |  |  |  |  |
|  | **SCI+ND Average** | 12.01 | 9.96 |  |  |  |  |  |  |  |  |  |  |  |
