# Supplementary material for: Novel stochastic framework for automatic segmentation of human thigh MRI volumes and its applications in spinal cord injured individuals
Source: PLoS One. 2019 May 9;14(5):e0216487. doi: 10.1371/journal.pone.0216487 (PMC6508923; doi:10.1371/journal.pone.0216487)
Supplement: S3 Table — Accuracy values for segmenting extensor, flexor and medial muscle compartments based on Dice similarity index (SI), Precision (P), Recall (R) and Hausdorff distance (HD) measures. (DOCX) [file pone.0216487.s003.docx]

**S3 Table.** **Accuracy values of ANTs method**. Accuracy values for segmenting extensor, flexor and medial muscle compartments based on Dice similarity index (SI), Precision (P), Recall (R) and Hausdorff distance (HD) measures.

| **SI (Dice)** | **SCI ID** | **EXTENSOR** | **FLEXOR** | **MEDIAL** | **ND ID** | **EXTENSOR** | **FLEXOR** | **MEDIAL** |
| --- | --- | --- | --- | --- | --- | --- | --- | --- |
|  | Subject 01 | 0.89 | 0.72 | 0.94 | Subject 01 | 0.93 | 0.87 | 0.81 |
|  | Subject 02 | 0.89 | 0.84 | 0.93 | Subject 02 | 0.93 | 0.87 | 0.89 |
|  | Subject 03 | 0.88 | 0.72 | 0.87 | Subject 03 | 0.94 | 0.85 | 0.89 |
|  | Subject 04 | 0.87 | 0.84 | 0.90 | Subject 04 | 0.89 | 0.87 | 0.77 |
|  | Subject 05 | 0.84 | 0.89 | 0.89 | Subject 05 | 0.97 | 0.92 | 0.94 |
|  | Subject 06 | 0.77 | 0.77 | 0.73 | Subject 06 | 0.79 | 0.75 | 0.82 |
|  | Subject 07 | 0.56 | 0.61 | 0.63 | Subject 07 | 0.83 | 0.87 | 0.77 |
|  | Subject 08 | 0.74 | 0.68 | 0.72 | Subject 08 | 0.87 | 0.89 | 0.88 |
|  | Subject 09 | 0.60 | 0.62 | 0.71 | Subject 09 | 0.69 | 0.63 | 0.66 |
|  | Subject 10 | 0.93 | 0.91 | 0.82 | Subject 10 | 0.97 | 0.94 | 0.96 |
|  | Subject 11 | 0.96 | 0.95 | 0.95 | Subject 11 | 0.96 | 0.94 | 0.95 |
|  | Subject 12 | 0.94 | 0.88 | 0.89 | Subject 12 | 0.79 | 0.75 | 0.82 |
|  | Subject 13 | 0.94 | 0.84 | 0.89 | Subject 13 | 0.93 | 0.87 | 0.81 |
|  | Subject 14 | 0.92 | 0.85 | 0.89 | Subject 14 | 0.96 | 0.94 | 0.95 |
|  | Subject 15 | 0.77 | 0.77 | 0.73 | **SD** | 0.08 | 0.09 | 0.09 |
|  | Subject 16 | 0.87 | 0.84 | 0.90 | **Averge** | 0.89 | 0.85 | 0.85 |
|  | **SD** | 0.12 | 0.10 | 0.10 | **Average ND** | 0.86 | 0.09 |  |
|  | **Averge** | 0.84 | 0.79 | 0.84 |  |  |  |  |
|  | **Average SCI** | 0.82 | 0.11 |  |  |  |  |  |
|  | **SCI+ND Average** | 0.84 | 0.10 |  |  |  |  |  |
| **Precision** | **SCI ID** | **EXTENSOR** | **FLEXOR** | **MEDIAL** | **ND ID** | **EXTENSOR** | **FLEXOR** | **MEDIAL** |
|  | Subject 01 | 0.96 | 0.95 | 0.95 | Subject 01 | 0.98 | 0.95 | 0.93 |
|  | Subject 02 | 0.97 | 0.97 | 0.92 | Subject 02 | 0.94 | 0.87 | 0.96 |
|  | Subject 03 | 0.97 | 0.96 | 0.95 | Subject 03 | 0.87 | 0.86 | 0.95 |
|  | Subject 04 | 0.96 | 0.96 | 0.87 | Subject 04 | 0.82 | 0.69 | 0.73 |
|  | Subject 05 | 0.97 | 0.99 | 0.86 | Subject 05 | 0.95 | 0.93 | 0.90 |
|  | Subject 06 | 0.92 | 0.90 | 0.99 | Subject 06 | 0.89 | 0.79 | 0.92 |
|  | Subject 07 | 0.97 | 0.90 | 0.80 | Subject 07 | 0.89 | 0.93 | 0.97 |
|  | Subject 08 | 0.96 | 0.73 | 0.86 | Subject 08 | 0.97 | 0.97 | 0.91 |
|  | Subject 09 | 1.00 | 0.82 | 0.87 | Subject 09 | 0.92 | 0.81 | 0.99 |
|  | Subject 10 | 1.00 | 0.97 | 0.71 | Subject 10 | 0.96 | 0.92 | 0.94 |
|  | Subject 11 | 0.95 | 0.98 | 0.96 | Subject 11 | 0.94 | 0.93 | 0.93 |
|  | Subject 12 | 0.94 | 0.85 | 0.93 | Subject 12 | 0.95 | 0.97 | 0.84 |
|  | Subject 13 | 0.92 | 0.90 | 0.91 | Subject 13 | 0.93 | 0.92 | 0.70 |
|  | Subject 14 | 0.89 | 0.91 | 0.97 | Subject 14 | 0.81 | 0.93 | 0.88 |
|  | Subject 15 | 0.97 | 0.72 | 0.80 | **SD** | 0.05 | 0.08 | 0.09 |
|  | Subject 16 | 0.90 | 0.80 | 0.93 | **Average** | 0.92 | 0.89 | 0.90 |
|  | **SD** | 0.03 | 0.09 | 0.07 | **Average ND** | 0.90 | 0.07 |  |
|  | **Average** | 0.95 | 0.89 | 0.89 |  |  |  |  |
|  | **Average SCI** | 0.91 | 0.07 |  |  |  |  |  |
|  | **SCI+ND Average** | 0.91 | 0.07 |  |  |  |  |  |
|  | **SCI ID** | **EXTENSOR** | **FLEXOR** | **MEDIAL** | **ND ID** | **EXTENSOR** | **FLEXOR** | **MEDIAL** |
| **Recall** | Subject 01 | 0.87 | 0.85 | 0.97 | Subject 01 | 0.98 | 0.95 | 0.96 |
|  | Subject 02 | 0.97 | 0.93 | 0.94 | Subject 02 | 0.99 | 0.80 | 0.99 |
|  | Subject 03 | 0.94 | 0.90 | 0.85 | Subject 03 | 0.99 | 0.95 | 0.81 |
|  | Subject 04 | 0.90 | 0.89 | 0.90 | Subject 04 | 1.00 | 0.82 | 0.68 |
|  | Subject 05 | 0.97 | 0.88 | 0.93 | Subject 05 | 0.98 | 0.91 | 0.97 |
|  | Subject 06 | 0.66 | 0.67 | 0.58 | Subject 06 | 0.72 | 0.71 | 0.74 |
|  | Subject 07 | 0.40 | 0.46 | 0.52 | Subject 07 | 0.79 | 0.81 | 0.63 |
|  | Subject 08 | 0.60 | 0.64 | 0.63 | Subject 08 | 0.79 | 0.82 | 0.85 |
|  | Subject 09 | 0.43 | 0.49 | 0.61 | Subject 09 | 0.55 | 0.52 | 0.49 |
|  | Subject 10 | 0.87 | 0.85 | 0.97 | Subject 10 | 0.98 | 0.96 | 0.97 |
|  | Subject 11 | 0.97 | 0.93 | 0.94 | Subject 11 | 0.98 | 0.96 | 0.97 |
|  | Subject 12 | 0.94 | 0.90 | 0.85 | Subject 12 | 0.98 | 0.97 | 0.89 |
|  | Subject 13 | 0.96 | 0.79 | 0.87 | Subject 13 | 0.92 | 0.82 | 0.95 |
|  | Subject 14 | 0.97 | 0.88 | 0.93 | Subject 14 | 0.72 | 0.71 | 0.74 |
|  | Subject 15 | 0.66 | 0.67 | 0.58 | **SD** | 0.14 | 0.13 | 0.15 |
|  | Subject 16 | 0.66 | 0.67 | 0.58 | **Average** | 0.88 | 0.84 | 0.83 |
|  | **SD** | 0.20 | 0.16 | 0.17 | **Average ND** | 0.85 | 0.14 |  |
|  | **Average** | 0.80 | 0.77 | 0.79 |  |  |  |  |
|  | **Average SCI** | 0.79 | 0.17 |  |  |  |  |  |
|  | **SCI+ND Average** | 0.82 | 0.16 |  |  |  |  |  |
| **HD** | **SCI ID** | **EXTENSOR** | **FLEXOR** | **MEDIAL** | **ND ID** | **EXTENSOR** | **FLEXOR** | **MEDIAL** |
|  | Subject 01 | 22.02 | 10.30 | 9.22 | Subject 01 | 15.33 | 10.25 | 19.85 |
|  | Subject 02 | 23.35 | 21.42 | 26.02 | Subject 02 | 4.47 | 9.64 | 40.91 |
|  | Subject 03 | 22.02 | 10.30 | 9.22 | Subject 03 | 14.04 | 16.58 | 24.78 |
|  | Subject 04 | 14.49 | 14.76 | 24.78 | Subject 04 | 23.11 | 11.05 | 28.62 |
|  | Subject 05 | 10.05 | 7.55 | 8.60 | Subject 05 | 26.63 | 26.65 | 29.22 |
|  | Subject 06 | 14.46 | 15.84 | 21.61 | Subject 06 | 21.77 | 18.06 | 23.60 |
|  | Subject 07 | 27.00 | 22.34 | 25.48 | Subject 07 | 15.33 | 10.25 | 26.08 |
|  | Subject 08 | 15.07 | 13.60 | 18.49 | Subject 08 | 13.00 | 9.95 | 55.52 |
|  | Subject 09 | 7.48 | 5.74 | 17.52 | Subject 09 | 26.63 | 26.65 | 29.22 |
|  | Subject 10 | 26.31 | 22.56 | 26.48 | Subject 10 | 12.37 | 17.46 | 24.35 |
|  | Subject 11 | 22.02 | 10.30 | 9.22 | Subject 11 | 14.04 | 16.58 | 26.48 |
|  | Subject 12 | 22.02 | 10.30 | 9.22 | Subject 12 | 22.02 | 10.30 | 27.28 |
|  | Subject 13 | 22.23 | 8.83 | 17.09 | Subject 13 | 15.65 | 15.75 | 42.54 |
|  | Subject 14 | 23.35 | 21.42 | 26.02 | Subject 14 | 9.49 | 10.05 | 41.88 |
|  | Subject 15 | 28.32 | 19.24 | 41.88 | **SD** | 6.48 | 5.91 | 9.93 |
|  | Subject 16 | 23.35 | 21.42 | 26.02 | **Average** | 16.70 | 14.94 | 31.45 |
|  | **SD** | 6.07 | 5.90 | 9.29 | **Average ND** | 21.03 | 10.57 |  |
|  | **Average** | 20.22 | 14.75 | 19.80 |  |  |  |  |
|  | **Average SCI** | 18.26 | 7.54 |  |  |  |  |  |
|  | **SCI+ND Average** | 19.64 | 9.05 |  |  |  |  |  |
