# Supplementary material for: Novel stochastic framework for automatic segmentation of human thigh MRI volumes and its applications in spinal cord injured individuals
Source: PLoS One. 2019 May 9;14(5):e0216487. doi: 10.1371/journal.pone.0216487 (PMC6508923; doi:10.1371/journal.pone.0216487)
Supplement: S4 Table — Accuracy values for segmenting extensor, flexor and medial muscle compartments based on Dice similarity index (SI), Precision (P), Recall (R) and Hausdorff distance (HD) measures. (DOCX) [file pone.0216487.s004.docx]

**S4 Table.** **Accuracy values of STAPLE method**. Accuracy values for segmenting extensor, flexor and medial muscle compartments based on Dice similarity index (SI), Precision (P), Recall (R) and Hausdorff distance (HD) measures.

| **SI (Dice)** | **SCI ID** | **EXTENSOR** | **FLEXOR** | **MEDIAL** | **ND ID** | **EXTENSOR** | **FLEXOR** | **MEDIAL** |
| --- | --- | --- | --- | --- | --- | --- | --- | --- |
|  | Subject 01 | 0.87 | 0.72 | 0.87 | Subject 01 | 0.97 | 0.94 | 0.94 |
|  | Subject 02 | 0.93 | 0.84 | 0.90 | Subject 02 | 0.96 | 0.92 | 0.92 |
|  | Subject 03 | 0.78 | 0.89 | 0.86 | Subject 03 | 0.93 | 0.90 | 0.88 |
|  | Subject 04 | 0.86 | 0.91 | 0.82 | Subject 04 | 0.89 | 0.87 | 0.77 |
|  | Subject 05 | 0.97 | 0.93 | 0.89 | Subject 05 | 0.97 | 0.92 | 0.94 |
|  | Subject 06 | 0.78 | 0.89 | 0.86 | Subject 06 | 0.82 | 0.76 | 0.85 |
|  | Subject 07 | 0.64 | 0.65 | 0.66 | Subject 07 | 0.84 | 0.90 | 0.79 |
|  | Subject 08 | 0.77 | 0.70 | 0.79 | Subject 08 | 0.87 | 0.91 | 0.89 |
|  | Subject 09 | 0.68 | 0.66 | 0.76 | Subject 09 | 0.73 | 0.87 | 0.72 |
|  | Subject 10 | 0.94 | 0.88 | 0.89 | Subject 10 | 0.97 | 0.94 | 0.96 |
|  | Subject 11 | 0.96 | 0.95 | 0.95 | Subject 11 | 0.82 | 0.76 | 0.85 |
|  | Subject 12 | 0.94 | 0.88 | 0.89 | Subject 12 | 0.82 | 0.76 | 0.85 |
|  | Subject 13 | 0.94 | 0.84 | 0.89 | Subject 13 | 0.93 | 0.87 | 0.81 |
|  | Subject 14 | 0.92 | 0.85 | 0.89 | Subject 14 | 0.97 | 0.92 | 0.94 |
|  | Subject 15 | 0.87 | 0.72 | 0.87 | **SD** | 0.08 | 0.07 | 0.07 |
|  | Subject 16 | 0.86 | 0.91 | 0.82 | **Average** | 0.89 | 0.87 | 0.86 |
|  | **SD** | 0.10 | 0.10 | 0.07 | **Average ND** | 0.88 | 0.07 |  |
|  | **Average** | 0.86 | 0.83 | 0.85 |  |  |  |  |
|  | **Average SCI** | 0.84 | 0.09 |  |  |  |  |  |
|  | **SCI+ND Average** | 0.86 | 0.08 |  |  |  |  |  |
| **Precision** | **SCI ID** | **EXTENSOR** | **FLEXOR** | **MEDIAL** | **ND ID** | **EXTENSOR** | **FLEXOR** | **MEDIAL** |
|  | Subject 01 | 0.96 | 0.95 | 0.95 | Subject 01 | 0.98 | 0.95 | 0.93 |
|  | Subject 02 | 0.97 | 0.97 | 0.92 | Subject 02 | 0.94 | 0.87 | 0.96 |
|  | Subject 03 | 0.97 | 0.96 | 0.95 | Subject 03 | 0.87 | 0.86 | 0.95 |
|  | Subject 04 | 0.96 | 0.96 | 0.87 | Subject 04 | 0.81 | 0.93 | 0.88 |
|  | Subject 05 | 0.97 | 0.99 | 0.86 | Subject 05 | 0.95 | 0.93 | 0.90 |
|  | Subject 06 | 0.95 | 0.87 | 0.98 | Subject 06 | 0.88 | 0.70 | 0.86 |
|  | Subject 07 | 0.97 | 0.92 | 0.77 | Subject 07 | 0.90 | 0.90 | 0.95 |
|  | Subject 08 | 0.97 | 0.75 | 0.88 | Subject 08 | 0.99 | 0.96 | 0.90 |
|  | Subject 09 | 1.00 | 0.83 | 0.85 | Subject 09 | 0.92 | 0.84 | 0.97 |
|  | Subject 10 | 1.00 | 0.97 | 0.71 | Subject 10 | 0.96 | 0.92 | 0.94 |
|  | Subject 11 | 0.95 | 0.98 | 0.96 | Subject 11 | 0.94 | 0.93 | 0.93 |
|  | Subject 12 | 0.94 | 0.85 | 0.93 | Subject 12 | 0.95 | 0.97 | 0.84 |
|  | Subject 13 | 0.92 | 0.90 | 0.91 | Subject 13 | 0.93 | 0.92 | 0.70 |
|  | Subject 14 | 0.89 | 0.91 | 0.97 | Subject 14 | 0.89 | 0.91 | 0.97 |
|  | Subject 15 | 0.97 | 0.72 | 0.80 | **SD** | 0.05 | 0.07 | 0.07 |
|  | Subject 16 | 0.90 | 0.80 | 0.93 | **Average** | 0.92 | 0.90 | 0.91 |
|  | **SD** | 0.03 | 0.09 | 0.08 | **Average ND** | 0.91 | 0.06 |  |
|  | **Average** | 0.96 | 0.90 | 0.89 |  |  |  |  |
|  | **Average SCI** | 0.91 | 0.07 |  |  |  |  |  |
|  | **SCI+ND Average** | 0.91 | 0.07 |  |  |  |  |  |
|  | **SCI ID** | **EXTENSOR** | **FLEXOR** | **MEDIAL** | **ND ID** | **EXTENSOR** | **FLEXOR** | **MEDIAL** |
| **Recall** | Subject 01 | 0.98 | 0.94 | 0.92 | Subject 01 | 0.98 | 0.95 | 0.96 |
|  | Subject 02 | 0.97 | 0.89 | 0.94 | Subject 02 | 0.99 | 0.80 | 0.99 |
|  | Subject 03 | 0.97 | 0.88 | 0.94 | Subject 03 | 0.99 | 0.95 | 0.81 |
|  | Subject 04 | 0.90 | 0.89 | 0.90 | Subject 04 | 1.00 | 0.82 | 0.68 |
|  | Subject 05 | 0.97 | 0.88 | 0.93 | Subject 05 | 0.98 | 0.91 | 0.97 |
|  | Subject 06 | 0.66 | 0.92 | 0.76 | Subject 06 | 0.77 | 0.83 | 0.85 |
|  | Subject 07 | 0.47 | 0.50 | 0.58 | Subject 07 | 0.79 | 0.90 | 0.67 |
|  | Subject 08 | 0.63 | 0.66 | 0.72 | Subject 08 | 0.77 | 0.86 | 0.88 |
|  | Subject 09 | 0.52 | 0.55 | 0.68 | Subject 09 | 0.61 | 0.90 | 0.57 |
|  | Subject 10 | 0.87 | 0.85 | 0.97 | Subject 10 | 0.98 | 0.96 | 0.97 |
|  | Subject 11 | 0.96 | 0.88 | 0.88 | Subject 11 | 0.96 | 0.93 | 0.96 |
|  | Subject 12 | 0.94 | 0.90 | 0.85 | Subject 12 | 0.98 | 0.97 | 0.89 |
|  | Subject 13 | 0.66 | 0.92 | 0.76 | Subject 13 | 0.98 | 0.96 | 0.97 |
|  | Subject 14 | 0.87 | 0.85 | 0.97 | Subject 14 | 0.92 | 0.82 | 0.95 |
|  | Subject 15 | 0.78 | 0.73 | 0.95 | **SD** | 0.12 | 0.06 | 0.14 |
|  | Subject 16 | 0.97 | 0.93 | 0.94 | **Average** | 0.91 | 0.90 | 0.87 |
|  | **SD** | 0.18 | 0.14 | 0.12 | **Average ND** | 0.89 | 0.11 |  |
|  | **Average** | 0.82 | 0.82 | 0.86 |  |  |  |  |
|  | **Average SCI** | 0.83 | 0.14 |  |  |  |  |  |
|  | **SCI+ND Average** | 0.86 | 0.13 |  |  |  |  |  |
| **HD** | **SCI ID** | **EXTENSOR** | **FLEXOR** | **MEDIAL** | **ND ID** | **EXTENSOR** | **FLEXOR** | **MEDIAL** |
|  | Subject 01 | 28.32 | 19.24 | 25.08 | Subject 01 | 12.37 | 17.46 | 24.35 |
|  | Subject 02 | 15.36 | 11.36 | 40.80 | Subject 02 | 15.65 | 15.75 | 42.54 |
|  | Subject 03 | 16.40 | 34.50 | 21.21 | Subject 03 | 10.05 | 14.46 | 25.32 |
|  | Subject 04 | 12.08 | 11.40 | 32.39 | Subject 04 | 23.11 | 11.05 | 28.62 |
|  | Subject 05 | 10.05 | 7.55 | 8.60 | Subject 05 | 4.12 | 7.35 | 26.08 |
|  | Subject 06 | 14.46 | 11.05 | 25.55 | Subject 06 | 18.87 | 20.15 | 30.55 |
|  | Subject 07 | 26.02 | 20.83 | 21.21 | Subject 07 | 15.65 | 9.11 | 18.60 |
|  | Subject 08 | 15.07 | 14.49 | 15.84 | Subject 08 | 13.75 | 16.31 | 55.52 |
|  | Subject 09 | 22.36 | 19.72 | 25.48 | Subject 09 | 26.31 | 22.56 | 26.48 |
|  | Subject 10 | 12.08 | 13.64 | 20.22 | Subject 10 | 6.16 | 10.49 | 36.36 |
|  | Subject 11 | 7.48 | 5.74 | 17.52 | Subject 11 | 9.27 | 14.87 | 32.59 |
|  | Subject 12 | 22.02 | 10.30 | 9.22 | Subject 12 | 14.04 | 16.58 | 20.35 |
|  | Subject 13 | 9.49 | 10.05 | 41.88 | Subject 13 | 6.40 | 14.14 | 59.51 |
|  | Subject 14 | 8.66 | 10.30 | 18.97 | Subject 14 | 4.12 | 7.35 | 26.08 |
|  | Subject 15 | 22.23 | 8.83 | 17.09 | **SD** | 6.79 | 4.58 | 12.31 |
|  | Subject 16 | 7.48 | 5.74 | 17.52 | **Average** | 12.85 | 14.12 | 32.35 |
|  | **SD** | 6.71 | 7.27 | 9.48 | **Average ND** | 19.77 | 12.28 |  |
|  | **Average** | 15.60 | 13.42 | 22.41 |  |  |  |  |
|  | **Average SCI** | 17.14 | 8.65 |  |  |  |  |  |
|  | **SCI+ND Average** | 18.46 | 10.46 |  |  |  |  |  |
