# Supplementary material for: Novel stochastic framework for automatic segmentation of human thigh MRI volumes and its applications in spinal cord injured individuals
Source: PLoS One. 2019 May 9;14(5):e0216487. doi: 10.1371/journal.pone.0216487 (PMC6508923; doi:10.1371/journal.pone.0216487)
Supplement: S6 Table — Calculated volumes and ratios for manual and automatic segmentation results for Extensor volume, Flexor volume, Medial volume, IMAT volume, SAT volume and Total muscle volume. (DOCX) [file pone.0216487.s006.docx]

**S6 Table.** **Comparison of automatic and manual segmentation**. Calculated volumes and ratios for manual and automatic segmentation results for Extensor volume, Flexor volume, Medial volume, IMAT volume, SAT volume and Total muscle volume.

| **CALCULATED VOLUMES (mm^3^) USING PROPOSED AUTOMATIC METHOD** | | | | | | | | | | | | | | | | |
| --- | --- | --- | --- | --- | --- | --- | --- | --- | --- | --- | --- | --- | --- | --- | --- | --- |
| **SCI ID** | **EXTENSOR** | **FLEXOR** | **MEDIAL** | **IMAT** | **SAT** | **MUSCLE** | **ND ID** | **EXTENSOR** | | **FLEXOR** | **MEDIAL** | **IMAT** | | **SAT** | | **MUSCLE** |
| Subject 01 | 411990 | 249892 | 345738 | 208751 | 687822 | 808427 | Subject 01 | 583851 | | 220995 | 617689 | 135162 | | 425706 | | 1227113 |
| Subject 02 | 303608 | 134521 | 191835 | 67453 | 335539 | 566838 | Subject 02 | 918581 | | 351317 | 499173 | 337102 | | 728666 | | 1354739 |
| Subject 03 | 340035 | 192125 | 280378 | 171406 | 518906 | 638135 | Subject 03 | 858792 | | 335944 | 408233 | 189128 | | 753516 | | 1330192 |
| Subject 04 | 148773 | 97234 | 118668 | 93427 | 740981 | 277307 | Subject 04 | 1160818 | | 328067 | 459159 | 211879 | | 772200 | | 1704274 |
| Subject 05 | 359188 | 233351 | 208619 | 144298 | 390920 | 650309 | Subject 05 | 519274 | | 204056 | 269477 | 120150 | | 335806 | | 794583 |
| Subject 06 | 392955 | 195301 | 297270 | 187626 | 667035 | 683124 | Subject 06 | 759787 | | 279804 | 387312 | 283001 | | 1222628 | | 1107425 |
| Subject 07 | 172803 | 107561 | 113407 | 79036 | 218916 | 304709 | Subject 07 | 967646 | | 301580 | 379603 | 233057 | | 844952 | | 1343655 |
| Subject 08 | 600710 | 152297 | 231879 | 221103 | 419178 | 769622 | Subject 08 | 900720 | | 334398 | 433698 | 228970 | | 1090398 | | 1329868 |
| Subject 09 | 478346 | 202554 | 253483 | 74517 | 268620 | 834145 | Subject 09 | 747036 | | 355107 | 449854 | 292096 | | 745031 | | 1162647 |
| Subject 10 | 373727 | 194430 | 252929 | 166644 | 166668 | 655749 | Subject 10 | 787914 | | 324915 | 438811 | 135216 | | 465710 | | 1348684 |
| Subject 11 | 266298 | 161538 | 183830 | 110001 | 421416 | 487580 | Subject 11 | 632013 | | 254451 | 397531 | 171261 | | 567149 | | 1055899 |
| Subject 12 | 183167 | 99449 | 160924 | 45403 | 130679 | 425373 | Subject 12 | 781093 | | 311573 | 383346 | 216101 | | 547108 | | 1206843 |
| Subject 13 | 468601 | 120968 | 383043 | 72544 | 454166 | 908528 | Subject 13 | 569514 | | 272275 | 323247 | 70723 | | 358172 | | 1052663 |
| Subject 14 | 284631 | 126174 | 170701 | 99799 | 1054434 | 499790 | Subject 14 | 730225 | | 283281 | 356144 | 82137 | | 727579 | | 1221423 |
| Subject 15 | 266572 | 174887 | 260023 | 112234 | 616760 | 622883 |  |  | |  |  |  | |  | |  |
| Subject 16 | 179467 | 75098 | 171957 | 43981 | 568771 | 418601 |  |  | |  |  |  | |  | |  |
| **p-values** | 6.5E-06 | 7.9E-06 | 1.7E-05 | 9.5E-03 | 2.3E-02 | 1.7E-05 |  |  | |  |  |  | |  | |  |
| **Decision** | TRUE | TRUE | TRUE | TRUE | TRUE | TRUE |  |  | |  |  |  | |  | |  |
| **CALCULATED VOLUMES (mm^3^) USING MANUAL METHOD** | | | | | | | | | | | | | | | | |
| **SCI ID** | **EXTENSOR** | **FLEXOR** | **MEDIAL** | **IMAT** | **SAT** | **MUSCLE** | **ND ID** | | **EXTENSOR** | **FLEXOR** | **MEDIAL** | | **IMAT** | | **SAT** | **MUSCLE** |
| Subject 01 | 406208 | 254144 | 357024 | 216304 | 657818 | 812403 | Subject 01 | | 698537 | 330926 | 407484 | | 119630 | | 413033 | 1251079 |
| Subject 02 | 303494 | 147076 | 187299 | 66096 | 344000 | 574422 | Subject 02 | | 808670 | 283260 | 638553 | | 294303 | | 682685 | 1429478 |
| Subject 03 | 340004 | 208551 | 284256 | 168787 | 521984 | 667805 | Subject 03 | | 692054 | 292292 | 566382 | | 144460 | | 754262 | 1393089 |
| Subject 04 | 158419 | 105415 | 114602 | 79201 | 756000 | 306487 | Subject 04 | | 947977 | 369816 | 592927 | | 205686 | | 762450 | 1706582 |
| Subject 05 | 356471 | 260678 | 193921 | 165510 | 413232 | 636400 | Subject 05 | | 506429 | 208278 | 249622 | | 98432 | | 309457 | 840753 |
| Subject 06 | 364105 | 205605 | 327250 | 196520 | 651277 | 721234 | Subject 06 | | 661979 | 281033 | 432867 | | 199007 | | 1208837 | 1189299 |
| Subject 07 | 179732 | 138507 | 88212 | 77996 | 226176 | 313446 | Subject 07 | | 762062 | 275842 | 559784 | | 231346 | | 845485 | 1347324 |
| Subject 08 | 456175 | 264249 | 294121 | 184727 | 485096 | 824303 | Subject 08 | | 824698 | 356839 | 451049 | | 165935 | | 1077857 | 1408789 |
| Subject 09 | 500651 | 266156 | 180782 | 107028 | 333909 | 802865 | Subject 09 | | 680994 | 297047 | 523932 | | 237681 | | 726978 | 1229047 |
| Subject 10 | 427032 | 223989 | 184744 | 134555 | 210549 | 700542 | Subject 10 | | 750165 | 269271 | 480965 | | 66616 | | 450590 | 1431689 |
| Subject 11 | 261323 | 169867 | 188477 | 139489 | 437474 | 475345 | Subject 11 | | 602272 | 245818 | 388145 | | 174771 | | 563406 | 1057155 |
| Subject 12 | 182738 | 93646 | 176511 | 56501 | 149614 | 409984 | Subject 12 | | 707528 | 341668 | 377632 | | 219780 | | 540297 | 1208604 |
| Subject 13 | 449511 | 136954 | 396956 | 76738 | 439895 | 906098 | Subject 13 | | 574334 | 304158 | 238667 | | 73697 | | 350463 | 1059534 |
| Subject 14 | 264290 | 144447 | 199172 | 88594 | 1084323 | 523682 | Subject 14 | | 600011 | 177140 | 539015 | | 80902 | | 707619 | 1230842 |
| Subject 15 | 329696 | 172238 | 219935 | 114620 | 639184 | 622981 |  | |  |  |  | |  | |  |  |
| Subject 16 | 187169 | 58264 | 205697 | 34078 | 577114 | 438156 |  | |  |  |  | |  | |  |  |
| **p-values** | 3.6E-06 | 8.6E-05 | 3.5E-05 | 5.9E-02 | 5.9E-02 | 4.4E-06 |  | |  |  |  | |  | |  |  |
| **Decision** | TRUE | TRUE | TRUE | FALSE | FALSE | TRUE |  | |  |  |  | |  | |  |  |
