# Supplementary material for: Novel stochastic framework for automatic segmentation of human thigh MRI volumes and its applications in spinal cord injured individuals
Source: PLoS One. 2019 May 9;14(5):e0216487. doi: 10.1371/journal.pone.0216487 (PMC6508923; doi:10.1371/journal.pone.0216487)
Supplement: S7 Table — Run-time values for the proposed segmentation framework on CPU and 3D CNN method on GPU. (DOCX) [file pone.0216487.s007.docx]

**S7 Table.** **Execution Run-time**. Run-time values for the proposed segmentation framework on CPU and 3D CNN method on GPU.

| **THE PROPOSED METHOD** | | | | | | | |
| --- | --- | --- | --- | --- | --- | --- | --- |
| **SCI ID** | **RUN TIME (s)** | **RUN TIME (m)** | **RUN TIME (h)** | **ND ID** | **RUN TIME (s)** | **RUN TIME (m)** | **RUN TIME (h)** |
| Subject 01 | 736 | 12.27 | 0.20 | Subject 01 | 716 | 11.94 | 0.20 |
| Subject 02 | 670 | 11.17 | 0.19 | Subject 02 | 749 | 12.48 | 0.21 |
| Subject 03 | 643 | 10.72 | 0.18 | Subject 03 | 725 | 12.08 | 0.20 |
| Subject 04 | 353 | 5.89 | 0.10 | Subject 04 | 751 | 12.52 | 0.21 |
| Subject 05 | 640 | 10.66 | 0.18 | Subject 05 | 688 | 11.47 | 0.19 |
| Subject 06 | 642 | 10.71 | 0.18 | Subject 06 | 722 | 12.03 | 0.20 |
| Subject 07 | 551 | 9.18 | 0.15 | Subject 07 | 733 | 12.22 | 0.20 |
| Subject 08 | 705 | 11.74 | 0.20 | Subject 08 | 733 | 12.22 | 0.20 |
| Subject 09 | 699 | 11.66 | 0.19 | Subject 09 | 726 | 12.10 | 0.20 |
| Subject 10 | 702 | 11.70 | 0.20 | Subject 10 | 723 | 12.05 | 0.20 |
| Subject 11 | 613 | 10.22 | 0.17 | Subject 11 | 710 | 11.84 | 0.20 |
| Subject 12 | 418 | 6.97 | 0.12 | Subject 12 | 709 | 11.82 | 0.20 |
| Subject 13 | 133 | 2.21 | 0.04 | Subject 13 | 692 | 11.53 | 0.19 |
| Subject 14 | 524 | 8.73 | 0.15 | Subject 14 | 710 | 11.84 | 0.20 |
| Subject 15 | 671 | 11.18 | 0.19 | **Sum** | 10089 | 168.15 | 2.80 |
| Subject 16 | 124 | 2.06 | 0.03 | **Mean** | 721 | 12.01 | 0.20 |
| **Sum** | 8824 | 147.07 | 2.45 | **SD** | 18 | 0.30 | 0.01 |
| **Mean** | 552 | 9.19 | 0.15 |  |  |  |  |
| **SD** | 196 | 3.26 | 0.05 |  |  |  |  |
| **Total Run-Time** | 18913 | 315.22 | 5.25 |  |  |  |  |
| **3-D CNN** | | | | | | | |
| **SCI ID** | **RUN TIME (s)** | **RUN TIME (m)** | **RUN TIME (h)** | **ND ID** | **RUN TIME (s)** | **RUN TIME (m)** | **RUN TIME (h)** |
| Subject 01 | 54 | 0.90 | 0.02 | Subject 01 | 73 | 1.22 | 0.02 |
| Subject 02 | 47 | 0.78 | 0.01 | Subject 02 | 77 | 1.28 | 0.02 |
| Subject 03 | 69 | 1.15 | 0.02 | Subject 03 | 77 | 1.28 | 0.02 |
| Subject 04 | 47 | 0.78 | 0.01 | Subject 04 | 84 | 1.40 | 0.02 |
| Subject 05 | 54 | 0.90 | 0.02 | Subject 05 | 55 | 0.92 | 0.02 |
| Subject 06 | 50 | 0.83 | 0.01 | Subject 06 | 79 | 1.32 | 0.02 |
| Subject 07 | 39 | 0.65 | 0.01 | Subject 07 | 84 | 1.40 | 0.02 |
| Subject 08 | 54 | 0.90 | 0.02 | Subject 08 | 85 | 1.42 | 0.02 |
| Subject 09 | 62 | 1.03 | 0.02 | Subject 09 | 77 | 1.28 | 0.02 |
| Subject 10 | 46 | 0.77 | 0.01 | Subject 10 | 70 | 1.17 | 0.02 |
| Subject 11 | 74 | 1.23 | 0.02 | Subject 11 | 74 | 1.23 | 0.02 |
| Subject 12 | 55 | 0.92 | 0.02 | Subject 12 | 84 | 1.40 | 0.02 |
| Subject 13 | 84 | 1.40 | 0.02 | Subject 13 | 62 | 1.03 | 0.02 |
| Subject 14 | 70 | 1.17 | 0.02 | Subject 14 | 77 | 1.28 | 0.02 |
| Subject 15 | 84 | 1.40 | 0.02 | **sum** | 1058 | 17.63 | 0.29 |
| Subject 16 | 78 | 1.30 | 0.02 | **mean** | 76 | 1.26 | 0.02 |
| **Sum** | 967 | 16.12 | 0.27 | **std** | 9 | 0.14 | 0.00 |
| **Mean** | 60 | 1.01 | 0.02 | **Training** | **RUN TIME (s)** | **RUN TIME (m)** | **RUN TIME (h)** |
| **SD** | 14 | 0.24 | 0.00 | Training #1 | 61719 | 1028.65 | 17.14 |
| **Total Run-Time** | 187174 | 3119.57 | 51.99 | Training #2 | 61709 | 1028.48 | 17.14 |
|  |  |  |  | Training #3 | 61721 | 1028.68 | 17.14 |
|  |  |  |  | **Sum** | 185149 | 3085.82 | 51.43 |
|  |  |  |  | **Mean** | 61716 | 1028.61 | 17.14 |
|  |  |  |  | **SD** | 6 | 0.11 | 0.00 |
